# New 1,2,3-Triazole-Coumarin-Glycoside Hybrids and Their 1,2,4-Triazolyl Thioglycoside Analogs Targeting Mitochondria Apoptotic Pathway: Synthesis, Anticancer Activity and Docking Simulation

**DOI:** 10.3390/molecules27175688

**Published:** 2022-09-03

**Authors:** Wael A. El-Sayed, Fahad M. Alminderej, Marwa M. Mounier, Eman S. Nossier, Sayed M. Saleh, Asmaa F. Kassem

**Affiliations:** 1 Department of Chemistry, College of Science, Qassim University, Buraidah 51452, Saudi Arabia; 2 National Research Centre, Photochemistry Department, Dokki, Giza 12622, Egypt; 3 National Research Centre, Department of Pharmacognosy, Giza 12622, Egypt; 4 Department of Pharmaceutical Medicinal Chemistry and Drug Design, Faculty of Pharmacy (Girls), Al-Azhar University, Cairo 11754, Egypt; 5 Chemistry Branch, Department of Science and Mathematics, Faculty of Petroleum and Mining Engineering, Suez University, Suez 43721, Egypt; 6 National Research Centre, Department of Chemistry of Natural and Microbial Products, Giza 12622, Egypt

**Keywords:** 1,2,3-triazoles, coumarin, 1,2,4-triazoles, anticancer, glycosides, molecular docking, EGFR, CDK-2

## Abstract

Toxicity and resistance to newly synthesized anticancer drugs represent a challenging phenomenon of intensified concern arising from variation in drug targets and consequently the prevalence of the latter concern requires further research. The current research reports the design, synthesis, and anticancer activity of new 1,2,3-triazole-coumarin-glycosyl hybrids and their 1,2,4-triazole thioglycosides as well as acyclic analogs. The cytotoxic activity of the synthesized products was studied against a panel of human cancer cell lines. Compounds **8**, **10**, **16** and **21** resulted in higher activities against different human cancer cells. The impact of the hybrid derivative **10** upon different apoptotic protein markers, including cytochrome c, Bcl-2, Bax, and caspase-7 along with its effect on the cell cycle was investigated. It revealed a mitochondria-apoptotic effect on MCF-7 cells and had the ability to upregulate pro-apoptotic Bax protein and downregulate anti-apoptotic Bcl-2 protein and thus implies the apoptotic fate of the cells. Furthermore, the inhibitory activities against EGFR, VEGFR-2 and CDK-2/cyclin A2 kinases for **8**, **10** and **21** were studied to detect the mechanism of their high potency. The coumarin-triazole-glycosyl hybrids **8** and **10** illustrated excellent broad inhibitory activity (IC_50_= 0.22 ± 0.01, 0.93 ± 0.42 and 0.24 ± 0.20 μM, respectively, for compound **8**), (IC_50_ = 0.12 ± 0.50, 0.79 ± 0.14 and 0.15± 0. 60 μM, respectively, for compound **10**), in comparison with the reference drugs, erlotinib, sorafenib and roscovitine (IC_50_ = 0.18 ± 0.05, 1.58 ± 0.11 and 0.46 ± 0.30 μM, respectively). In addition, the docking study was simulated to afford better rationalization and put insight into the binding affinity between the promising derivatives and their targeted enzymes and that might be used as an optimum lead for further modification in the anticancer field.

## 1. Introduction

Cancer is still one of the major threats and, indeed, its complicated and heterogeneous nature is still associated with high mortality and morbidity rates. Consequently, and due to the gained resistance and toxicity against the current anticancer drugs, there is a great need to develop newer anticancer agents. Previous evidence indicates the importance of apoptosis in determining the cancer fate, as it considers as a key target for the discovery of novel anticancer candidates. Therefore, targeting the apoptotic signaling pathway by anticancer drugs is an important mechanism in cancer therapy [1]. There are numerous studies that have shown that there is a close link between mitochondria and cancer development [2]. As mitochondria play a key role in cell proliferation and death, many studies have demonstrated that targeting mitochondria can inhibit tumor growth or induce apoptosis [3]. Targeted chemotherapy involves several approaches, including selective enzyme inhibition, which has been recognized as an attractive strategy for inhibiting tumour growth [4,5,6,7]. Among the widely studied molecular targets are EGFR, VEGFR-2 and CDK-2 kinases that have a vital role in adjusting various cellular pathways as differentiation, migration, proliferation and angiogenesis [7].

The epidermal growth factor receptor (EGFR) tyrosine kinase controls many bioactivities, such as regulation of the cell cycle, adhesion and cell motility [8]. Therefore, over-expression or mutations of EGFR stimulate cell angiogenesis, proliferation, metastasis and anti-apoptosis affording different epidermal carcinomas, especially colon, breast and bladder cancers [9,10,11,12]. Vascular endothelial growth factor (VEGF) tyrosine kinase receptor is a family of the most crucial and specific signaling proteins involved in both pathological and physiological angiogenesis [13]. This family includes three protein receptors; VEGFR-1 and VEGFR-2 facilitate angiogenesis while VEGFR-3 mostly controls lymphangiogenesis [14,15,16]. Moreover, cyclin-dependent kinases (CDKs) are members of protein kinases that have an essential role in regulation of the sequential stages in the cell cycle [17]. The member, CDK-2, shares progression of cell cycle from the S-phase to the late G1-phase with initiation of DNA repair [18]. Therefore, downregulation or blocking of the signaling of these kinases has developed a major approach for the discovery of new cancer therapies [19].

**Figure 1:**
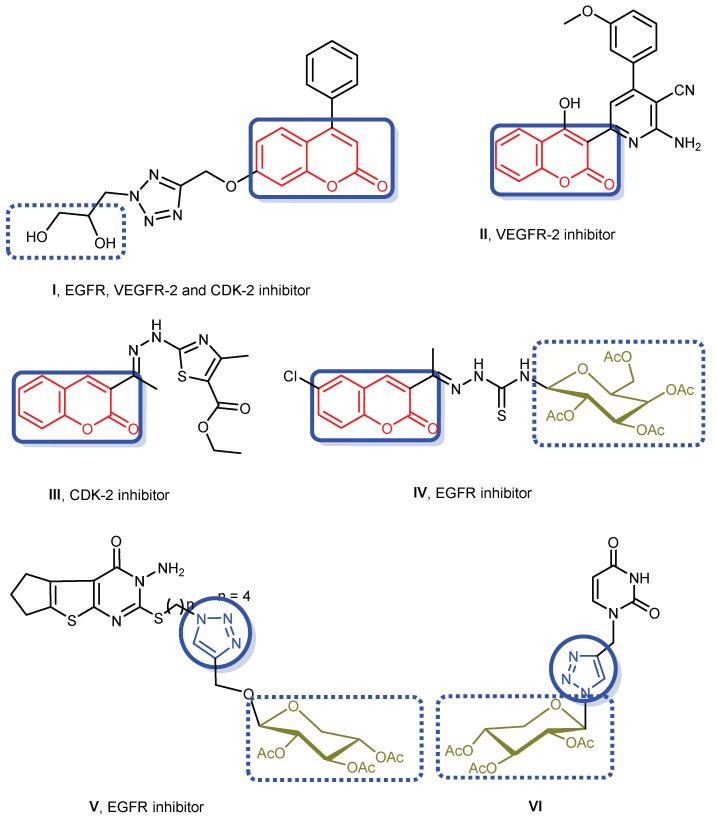
Some reported anticancer agents bearing coumarin, triazole and/or glycoside moieties with various mechanisms.

Coumarin is a potent inhibitor for many protein functions and enzymes and its derivatives have been widely reported to have multiple therapeutic applications like antitumor, anti-inflammatory, anti-allergic, antimicrobial, anti-viral, anti-HIV, anti-coagulant, and antioxidant agents [20,21,22,23]. Coumarin derivatives have not only been applied to treat cancer but also diminish the side effects from radiotherapy [24]. Recently, agents bearing coumarin nucleus I–IV have been well known and illustrated promising cytotoxic activity through inhibition of EGFR, VEGFR-2 and/or CDK-2 kinases as mentioned in Figure 1 [25,26,27,28].

Triazoles have become considerable back-bones in medicinal chemistry regarding its numerous pharmacological activities such as antioxidant, antitubercular, antibacterial, antiviral, anti-inflammatory and anticancer activities [29,30]. 1,2,3-Triazole is a privileged core that is prepared through catalyzed 1,3-dipolar cycloaddition reaction between terminal alkynes and azides as namely “Click-reaction” [11]. The generated triazole ring, via click chemistry, is capable of interplaying with biomolecular targets due to its high dipole moment and the adequacy for interesting hydrogen bond formations. Furthermore, 1,2,3-triazoles have been found stable toward metabolic degradation, and their existence in molecules could be useful in improving solubility and bioavailability which further favors their advantage as pharmacophoric moieties [31].

Additionally, many research studies have reported the antitumor potency of triazole containing compounds as **V** and **VI** (Figure 1) [11]. On the other hand, incorporation of heterocyclic compounds with glycosides has resulted in the creation of crucial hybrids that have gained great biological interest as anticancer, antimicrobial and antiviral activities [32,33,34,35,36,37,38]. Among them, compounds having heterocycle-glycoside motifs **IV**–**VI** have been reported with potent anticancer and inhibitory activity against EGFR [11,26] (Figure 1).

**Figure 2:**
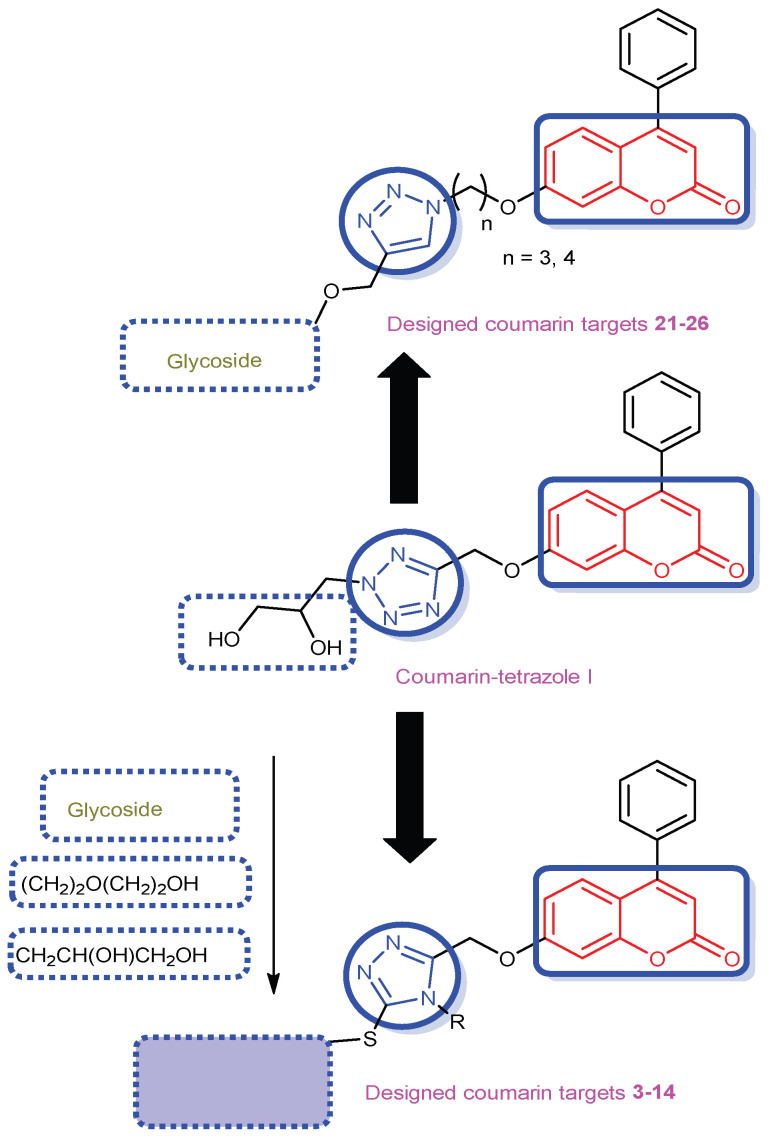
Design of new coumarin-triazole hybrids expected to possess potent antiproliferative activity through inhibition of EGFR, VEGFR-2 and CDK-2 kinases.

Considering the facts mentioned above and our recently reported coumarin-tetrazole analog **I,** in this present study, two sets of coumarin-triazole analogs were designed through bioisosterism and molecular hybridization with glycosides or other fragments through methyloxy or thio-linker (Figure 2). All the newly synthesized coumarin-triazole derivatives were evaluated as antiproliferative agents against different human cancer cell lines. The promising coumarin-triazole targets **8**, **10** and **21** were further screened for their kinase inhibitory assessment against EGFR, VEGFR-2 and CDK-2/cyclin A2 to determine their mechanism of action. The highly potent coumarin-triazole-glycoside **10** was subjected for cell cycle analysis and apoptosis as well as its impact upon cytochrome c, Bcl-2, Bax, and caspase-7 levels. As a final point, molecular docking study was furnished to detect a better rationalization and set insight to the binding modes between the targeted enzymes and the promising derivatives.

## 2. Results and Discussion

### 2.1. Chemistry

In the current research work, the targeted coumarin-azole-sugar hybrid compounds were synthesized via different strategies according to the type of the azolyl system linked to the coumarin system via methyl-oxy linkage. Glycosylation of the coumarin-triazole hybrid molecule, or a heterocyclization process affording the glycosyl-triazole, were applied resulting in the formation of the glycoside products and their analogs.

The first system is coumarin-1,2,4-triazole-thioghycoside hybrids which were prepared via glycosylation reaction of the starting *N^1^*-alkylated coumarin-1,2,4-triazole derivative (**1a,b**) [39] with the acetylated bromogalactopyranosyl or xylopyranosyl reagent **2a,b**, affording the corresponding 1,2,4-triazole thioglycosides **3**–**6**, in 70–73% yields, respectively. The ^1^H NMR spectra of the thioglycoside derivative **3** as a representative example displayed the characterizing signals of the protons of the CH_3_ in the acetyl groups at δ 1.94–1.97 ppm and the glycosyl moiety at δ 3.50–5.50 ppm, which has also been confirmed by its ^13^C NMR revealing the carbon signals of pyranosyl moiety at δ 61.7–92.1 ppm. The δ value of the signal assigned for the H^1^ anomeric at δ 5.52–5.75 ppm as well as its corresponding *J* coupling *J* = 9.8 Hz indicated the glycosylthio-attachment nature in addition to the *β*-configuration of the glycosyl moiety linkage. The attachment was confirmed by the fact that the H^1^ (anomeric) signal in glycosyl heterocycles incorporating a thione system with –NH- neighboring to C=S system, in which the glycopyranosyl moiety is attached to a nitrogen atom, was reported to be displayed at higher shifts due to the anisotropic deshielding effectiveness of such thione group [40]. 

**Scheme 1:**
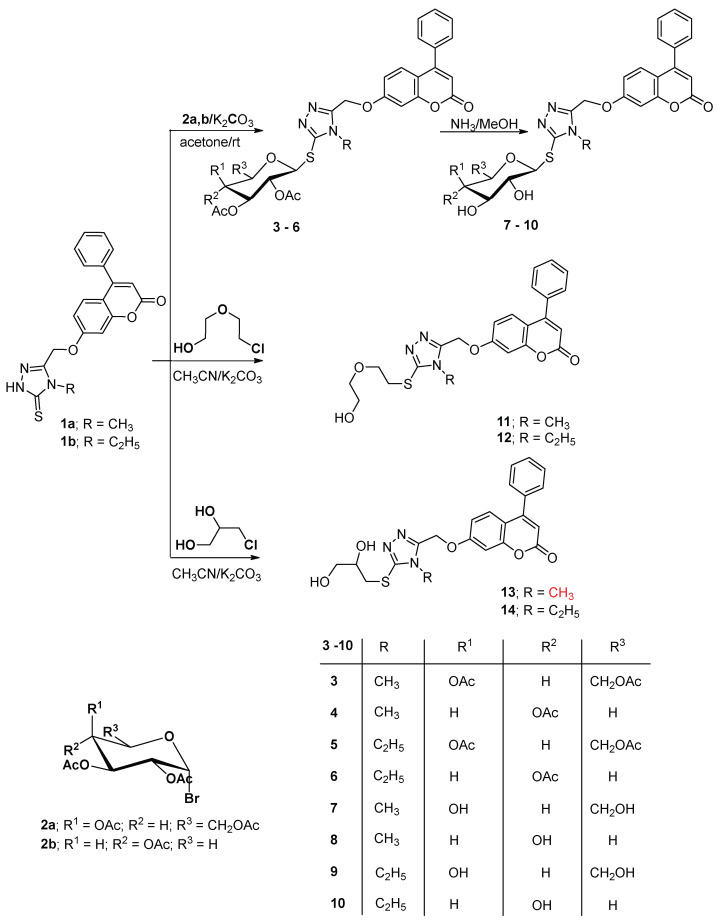
Synthesis of 1,2,4-triazole-coumarin hybrid thioglycosides.

Conversion of the attached *O*-acetyl groups in the acetylated thioglycosides **3**–**6** to their corresponding free hydroxyl deprotected thioglycosides **7**–**10**, respectively, was achieved by methanolic ammonia solution in good yields (Scheme 1). The well characterizing absorption bands assigned for the hydroxyl groups in the IR spectra of compound **10** at 3405–3425 cm^−1^ and the disappearance of the C=O band present at 1735 cm^−1^ in its acetylated precursor **6** confirmed the deacetylation reaction. Additionally, the assigned peaks of the acetyl-methyl groups in the acetylated products were gone in the ^1^H NMR spectra of the derived deprotected products and instead the signals of the hydroxyl groups appeared.

Alkylation of the starting coumarin-1,2,4-triazole derivatives **1a,b** with oxygenated and hydroxyl substituted alkyl halides was also carried out with the aim to get acyclic sugar analogues of the triazole glycosides. Thus, reaction of **1a,b** with 2-(2-chloroethoxy)ethan-1-ol) afforded the thio-oxygenated alkyl-1,2,4-triazole derivatives 11 and 12, respectively, while reaction with 1-chloro-2,3-dihydroxypropane resulted in the formation of compounds **13** and **14**, respectively. The nature of the side chain attachment to the triazole ring was confirmed by the NMR and IR spectra of the afforded products which revealed the presence of the signals of the hydroxyl and the methylene protons in the linked functionalized alkyl side chain. The alkylation center in structure **11**–**14** was also confirmed at the sulfur of the thiol compounds as the C=S signal was absent in their ^13^C NMR spectra and also the low chemical shift of CH_2_ attached to the sulfur atom at 3.05–3.33 ppm. The ^1^H NMR of the substituted triazole **14**, as a representative example, revealed the CH_2_ groups in the attached side chain at 3.05, 3.18, 3.45 and 4.09 as well as the hydroxyl groups at 5.21 and 5.56 ppm.

**Scheme 2:**
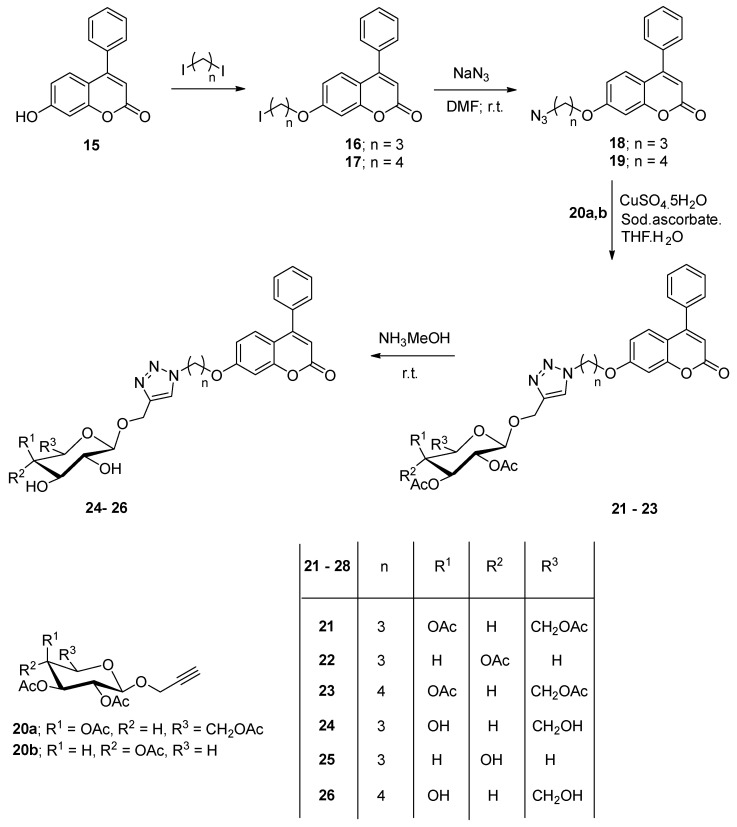
Synthesis of 1,2,3-triazol-coumarin hybrid glycosides.

The set of coumarin-azole-glycosyl hybrid compounds incorporates the 1,2,3-triazole system linking the substituted coumarin and the sugar moieties in which the glycon part was linked to the triazole ring via a C-C linkage forming products as analogs of *C*-nucleosides. The strategy of click dipolar cycloaddition (copper-catalyzed alkyn-azide; CuCAA) was applied for affording the glycopyranosyl-1,2,3-triazole-coumarin hybrids. Formation of the functionalized azides required for the cycloaddition process was performed via alkylation of 7-hydroxycpumarin (**15**) with 1,3-diiodopropane and its diiodobutane analogue to form the halogenated alkyl-methoxy coumarin derivatives **16** and **17**, respectively. The latter products were transformed into the corresponding azide derivatives **18** and **19**, respectively, by reaction with sodium azide. The resulting coumarin azide derivatives were efficiently applied in the preparation of the desired 1,2,3-triazole-*C*-glycosides based coumarin system by the click dipolar cycloaddition reaction using the appropriate terminal acetylenic sugar **20a,b** incorporating the 2,3,4,6-tetra-*O*-acetyl-D-galactopyranosyl or 2,3,4-tri-*O*-acetyl-D-xylopyranosyl moieties as alkyne reagents under click conditions, in 76–77% yields. The desired Cu(I) catalytic species were generated in-situ from sodium ascorbate and copper sulfate by in-situ reduction process of the C(II) ions. A mixture of tetrahydrofuran/water (2:1) was efficiently utilized as a mixed solvent system after investigating different systems. The ^1^H NMR spectra of the *C*-glycosyl-1,2,3-triazoles based coumarin displayed the hydrogen peaks of the glycopyranosyl part accounting for those of the CH_3_ in the acetyl groups and the other hydrogens of the galacto- and xylopyranosyl moieties. The latter structural confirmation was also achieved by the ^13^C NMR spectra which revealed all carbon signals at their characteristic positions. The *J*-value (9.8–10.2 Hz) concerning the anomeric hydrogen (*H^1^* in the sugar part) that has been displayed as a doublet signal indicated the configuration type of the attachment of the glycopyranosyl part to the substituted 1,2,3-triazole ring as a *β*-conformation. The acetylated 1,2,3-triazole glycosides based coumarin system (**21**–**23)** were deacetylate by using methanol saturated with gaseous ammonia and led to the formation of the derived 1,2,3-triazole-*C*-glycosides **24**–**26**, respectively possessing a sugar part with their free hydroxyl groups, respectively. The characteristic IR spectra for C=O signals in the acetyl groups of the starting protected glycoside **22**, as an example, at 1752 cm^-1^ were disappeared in its derived deacetylated product **25** and the hydroxyl absorption band clearly appeared at 3431-3420 cm^-1^**,** which affirmed the deacetylation reaction. Moreover, the glycosyl-acetyl signals were gone in the NMR spectra while those attributed to the hydroxyl protons were existed at 4.34, 4.70 and 5.01 for the deacetylated glycoside **25** as a representative example which is in agreement with the structures of the glycoside compounds with free hydroxyl sugar moieties (Scheme 2).

### 2.2. Biological Evaluation

Mitochondria represent an important organelle that participates in various physiological functions in eukaryote cells. Dysfunction of the mitochondria is the main lineament of tumor cells that prevents tumor cells from apoptosis [41]. Bcl-2 family proteins regulate the apoptosis pathway of mitochondria. The latter takes mitochondrial depolarization as a starting point, which is regulated by members of the Bcl-2 protein family, followed by the release of the apoptosis signal [42]. The substantial mitochondria apoptotic pathway is induced by various types of non-receptor-mediated stimuli which generate intracellular signals that target the cell apoptosis. These stimuli modify the mitochondrial membrane leading to the release of pro-apoptotic proteins which signal the execution pathway including caspase proteins leading to apoptosis.

#### 2.2.1. In Vitro Cytotoxicity Using MTT Assay

The synthesized compounds were studied for their cytotoxic activity against several human cancer cell lines, namely human osteosarcoma (HOS cell lines), human breast adenocarcinoma (MDA-MB-231 cell lines), human breast cancer (MCF-7 cell lines), human colorectal adenocarcinoma (Caco-2 cell lines) and human colon cell line (HCT-116 cell lines) using MTT assay at 100 µM, and the results were outlined as shown in Table 1. 

**Figure 3:**
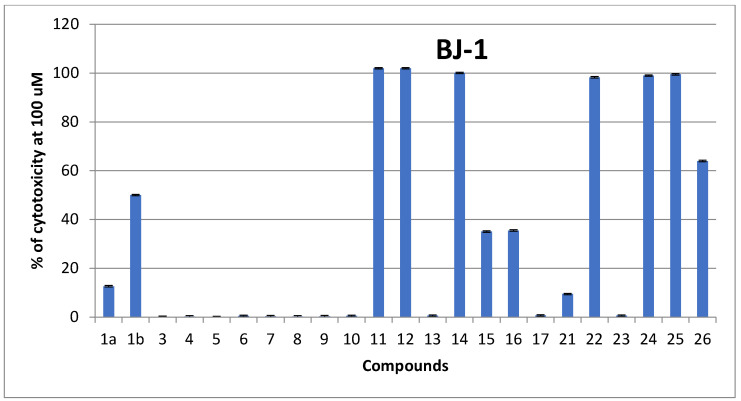
In vitro cytotoxic screening (%) of the newly synthesized coumarin derivatives upon normal human cell lines (BJ-1) at 100 µM; each result is a mean of three replicate samples, and values are represented as % inhibition.

To determine the selectivity and safety, all tested compounds were subjected to an assay for their safety upon normal human skin cell lines BJ-1 at a concentration of 100 µM. According to Figure 3, compounds **1b**, **11**, **12**, **14**, **22**, **24**, **25** and **26** possessed high toxicity on normal skin cells and therefore were excluded. The remaining safe promising derivatives that gave ≥60% cytotoxic activity upon their corresponding cells in a dose-dependent manner at concentrations ranging from (0.78–100 µM) were further screened to calculate their IC_50_ values as shown in Table 2.

According to the afforded results, compounds **8**, **10**, **15**, **16** and **21** exhibited promising and selective anticancer activity. By comparing their IC_50_ values, the most potent and safe coumarin-triazole **10** revealed a significant cytotoxic activity against MCF-7 cells with IC_50_ 19.6 µM. Hence, we decided to elucidate its anticancer effect over different apoptotic protein markers, including cytochrome c, Bcl-2, Bax and caspase-7 along with cell cycle analysis.

##### Structure Activity Relationship (SAR)

A possible correlation of the afforded cytotoxic activity results with the characteristic structural features of the tested compounds was performed. Generally, through inspection of the %cytotoxicity results against cancer cell lines, it was observed that the coumarin derivatives bearing the 1,2,3-triazole core **21**–**26** displayed higher cytotoxic activities than those having 1,2,4-triazole system **3**–**14**. In addition, the coumarin-1,2,4-triazole-thioglycosides incorporating the xylopyranosyl glycosyl moiety resulted in higher activity than those given by their galacopyranosyl analogues. The loss of the glycosyl moieties in 1,2,4-triazole derivatives **11**, **12** and **14** increased potencies against cancerous cell lines but revealed a higher toxicity against normal BJ-1 cell lines. For the coumarin-1,2,3-triazole-glycosyl hybrids, the chain length between coumarin and triazole moieties affected the potency. The coumarin based 1,2,3-triazole glycosides possessing propyloxy linker (*n* = 3) in compounds **21**, **22**, **24** and **25** revealed higher cytotoxic activities than their analogues with longer linker (butyloxy linker, *n* = 4) in the triazole glycosides **23** and **26**. Furthermore, the 1,2,3-triazole derivatives **24**–**26** bearing the glycosyl part with free hydroxyls exhibited superior potency than those with acetylated ones **21**–**23**, although the acetylated glycosides were less toxic and safer against normal cells.

From the previous observation, the cytotoxic activity may be attributed to the nature of the triazole and the glycoside moieties in addition to the chain length of linkers between the coumarin and triazole scaffolds. Concerning the cytotoxicity against cancerous cells and safety properties against normal cells, the coumarin-1,2,4-triazole-thioglycoside hybrid compound **10** was selected as a promising target for further screening to detect its possible mechanism of anticancer action.

#### 2.2.2. Effect on the Level of Bax, Bcl-2, Cytochrome c and Caspase-7 Protein in MCF-7 Cells

**Figure 4:**
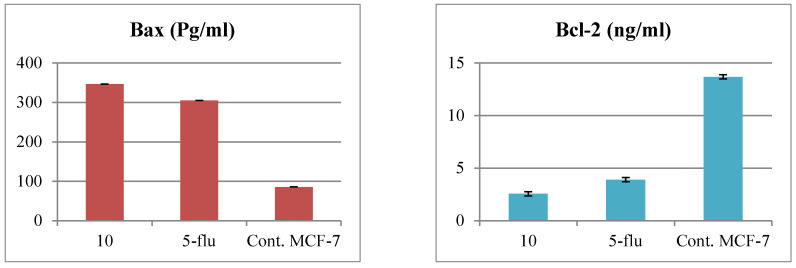

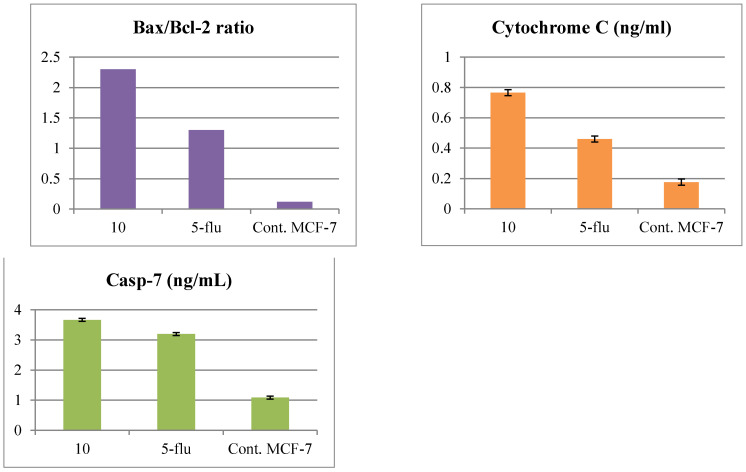
Levels of Bax, Bcl-2, Bax/Bcl-2 ratio, cytochrome c and caspase-7 protein in MCF-7 cells after treatment with compound **10** and the standard drug for 24 h compared with the untreated one, 5-flu: 5-fluorouracil as a reference drug.

Referring to the cytotoxicity results, the 1,2,4-triazole-thioglycoside derivative **10** has a mitochondria-apoptotic effect on breast cancer cells through reduction of MTT by a mitochondrial enzyme. Further studies were conducted on treating MCF-7 breast cancer cells with IC_50_ value (19.6 µM) of compound **10** for 24 h and determining its effect upon different apoptotic proteins. It was revealed that compound **10** upregulated the pro-apoptotic Bax protein by 4-folds and downregulated the anti-apoptotic Bcl-2 protein by 5.3-folds comparing with untreated cells with disruption of the Bax/Bcl-2 ratio indicative of the apoptotic fate of the cells. Along with that, such derivative elevated the release of cytochrome c into the cytosol in the treated MCF-7, in comparison with untreated one from 0.176 to 0.766 ng/ml due to disturbing mitochondrial outer membrane permeability (MOMP). In addition, the target **10** achieved better results than those afforded by from the standard 5-fluorouracil. Moreover, the level of caspase-7 was elevated in treated cells with the target **10** rather than that with 5-fluorouracil or the untreated cells as shown in Figure 4.

#### 2.2.3. Cell Cycle Arrest

**Figure 5:**
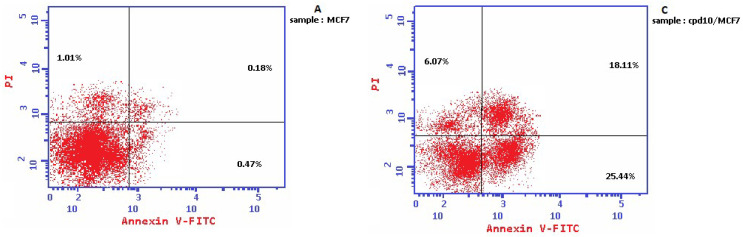

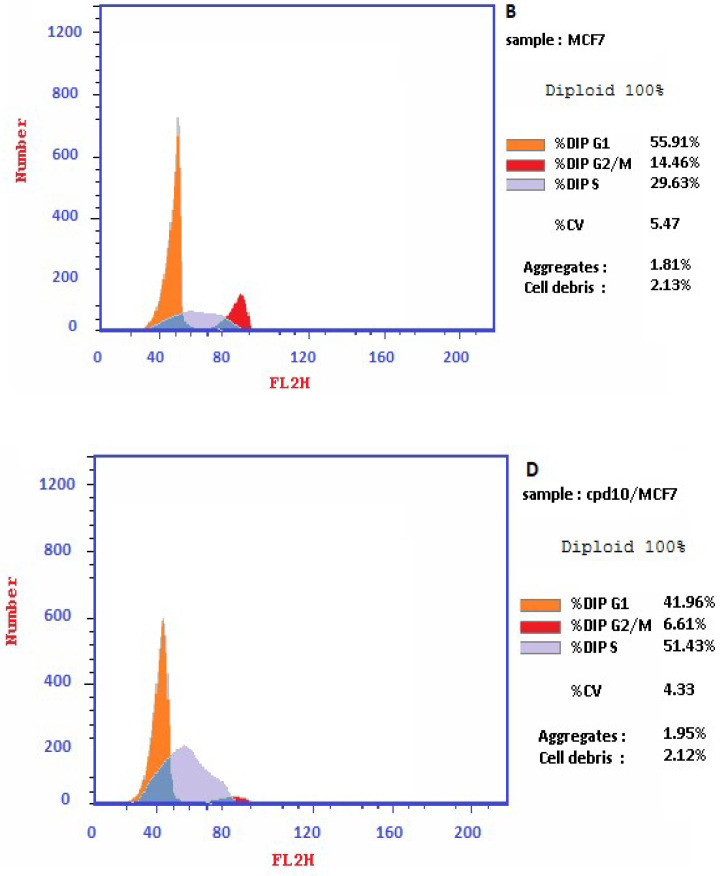
Cell cycle analysis and the effect of the target **10** on the percentage of V-FITC-positive annexin staining in MCF-7 cells in comparison with the control.

The results illustrated in Figure 5 displayed that the coumarin-1,2,4-triazole-thioglycoside **10** caused induction of the early apoptosis phase from 0.47 to 25.44% along with their elevation of secondary late apoptosis from 0.18 to 18.11% confirming the apoptotic effect of that compound. The molecular mechanism of action of compound **10** was studied upon treatment of MCF-7 cells with IC_50_ of 19.6 µM for 24 h using flow cytometry. Cell cycle analysis revealed a significant increase in the cell percentage at the S phase by 1.7 fold from 29.63% in untreated MCF-7 cells to 51.43% in treated ones. These results confirmed the apoptosis inside MCF-7 cells and cell cycle arrest at S phase.

#### 2.2.4. Kinase Inhibitory Assessment against EGFR, VEGFR-2 and CDK-2

The three most potent antiproliferative derivatives in coumarin-triazole series **8**, **10** and **21** were further screened for their inhibitory activity against EGFR, VEGFR-2 and CDK-2/cyclin A2 kinases to determine the mechanism of their promising anticancer activity. Their IC_50_ values were estimated in μM comparing with the references erlotinib, sorafenib and roscovitine, respectively [43,44,45], and depicted in Table 3.

By inspection of the obtained results, it was noted that the coumarin-triazole-glycosides **8** and **10** exhibited the highest kinase inhibitory activity against all screened enzymes (IC50 = 0.22 ± 0.01, 0.93 ± 0.42 and 0.24 ± 0.20 μM, respectively, for compound **8**), (IC50 = 0.12 ± 0.50, 0.79 ± 0.14 and 0.15± 0. 60 μM, respectively, for compound **10**), in comparison with the standards, erlotinib, sorafenib and roscovitine (IC50 = 0.18 ± 0.05, 1.58 ± 0.11 and 0.46 ± 0.30 μM, respectively). On the other hand, the analog **21** afforded probably no inhibitory activities against all screened enzymes and could exert its cytotoxicity through another mechanism of action.

The aforementioned data revealed that the coumarin-triazole-glycosides **8** and **10** could be attributed as multi-targeted inhibitors against EGFR, VEGFR-2 and CDK-2/cyclinA2 kinases. The weak potency of the coumarin-triazole-glycoside **21** was observed upon chain elongation between the coumarin and triazole moieties.

### 2.3. Molecular Docking Study

The promising kinase inhibitory results of the coumarin-triazole-glycoside targets **8** and **10** potentiate us to carry out the molecular docking to study their binding orientation within the active sites of EGFR, VEGFR-2 and CDK-2/cyclin A2 kinases (PDB codes: 1M17, 4ASD and 3DDQ, respectively) [45,46,47,48]. Validation of the docking processes using MOE-Dock software (Molecular Operating Environment) version 2014.0901 [49,50,51] was done through re-docking of the original ligands erlotinib, sorafenib and roscovitine within the binding sites of EGFR, VEGFR-2 and CDK-2/cyclin A2, revealing energy scores of −11.75, −10.42 and −11.36 kcal/mol with small RMSD values of 0.74, 1.33 and 0.81 Å, respectively, between the co-crystallized ligands and their docked poses. As reported, binding of the original ligands with the essential amino acids as Met769 though H-bonding in EGFR, Glu885, Cys919, Asp1046 and Phe1047 through H-bonding and arene–cation interactions in VEGFR-2 and Glu81, Leu83, Ile10 through H-bonding and arene–cation interactions in CDK-2, is considered as key interactions that help in obtaining the best score values [45,46,47,48].

**Figure 6:**
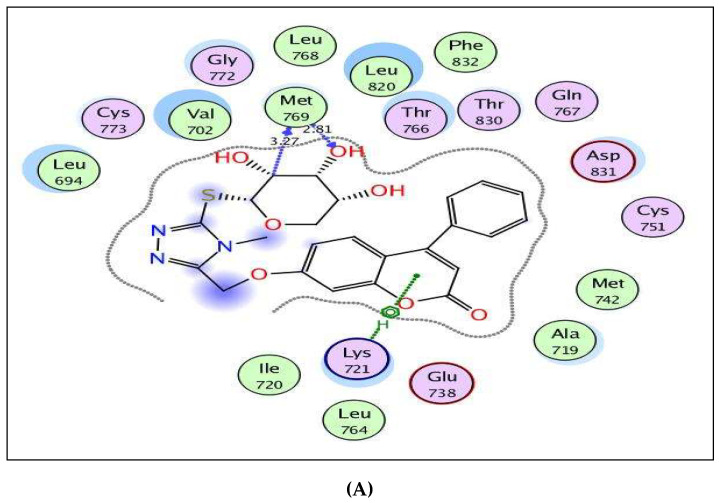

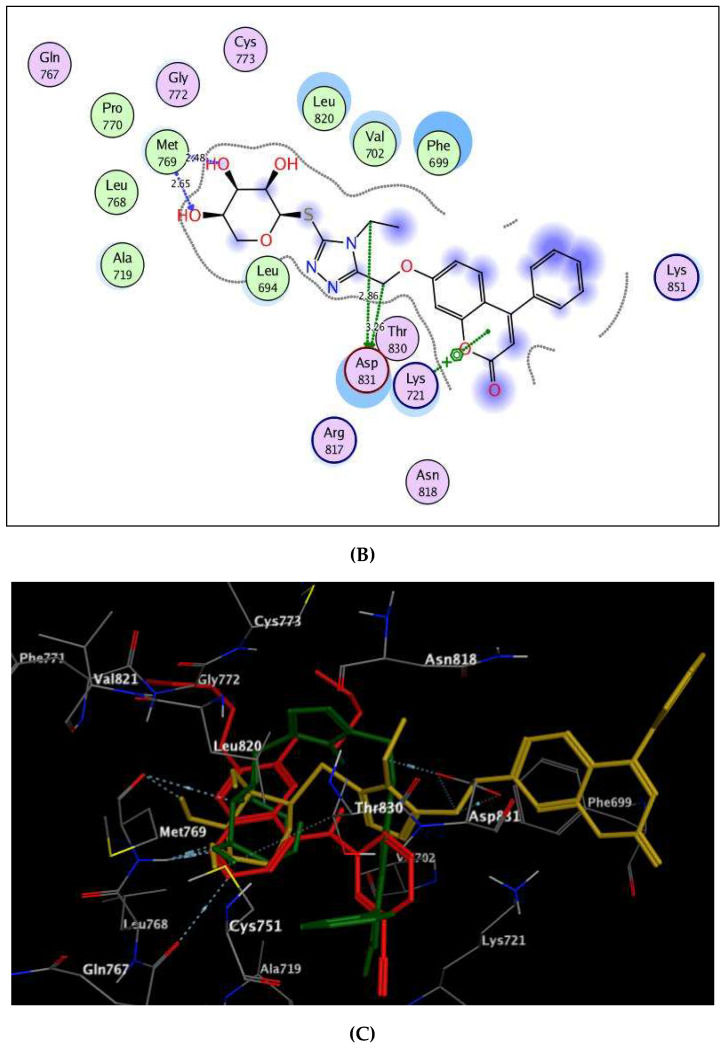
(**A**,**B**) views illustrate the 2D binding features of the coumarin targets, **8** and **10**, while (**C**) view illustrates the 3D superimposition between original ligand erlotinib (red), **8** (green) and **10** (yellow) within the active site of EGFR (PDB code: 1M17), respectively.

By focusing upon Figure 6 showing the binding site of EGFR, the coumarin-triazole derivatives **8** and **10** were fitted well through arene–cation interaction between the coumarin scaffold and Lys721. In addition, the glycoside moiety exhibited H-bond interactions with the backbone of the key amino acid, Met769, resembling the reference ligand, erlotinib. The inhibitory potency of coumarin-triazole-glycoside **10** was potentiated through two H-bond donors between the ethyl group at p-4 of thioxotriazole moiety and methoxy group at p-7 of coumarin with the sidechain of the essential amino acid Asp831 (distance: 2.86, 3.26 Å, respectively). Both targets **8** and **10** yielded good energy scores of −0.22 and −11.60 kcal/mol, respectively.

**Figure 7:**
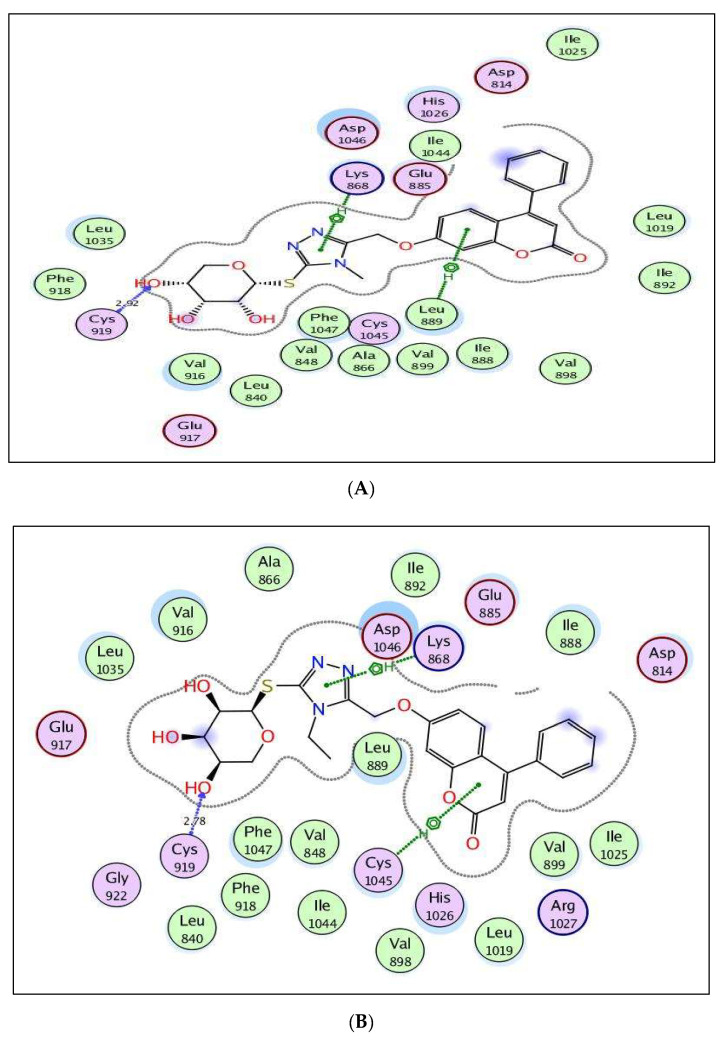

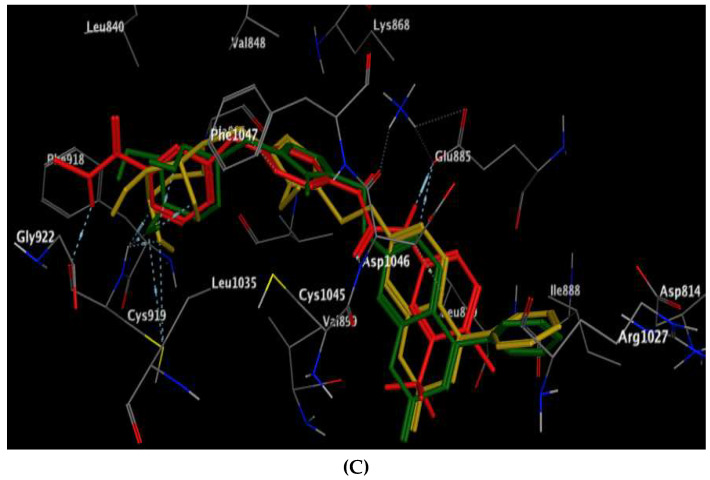
(**A**,**B**) views illustrate the 2D binding features of the coumarin targets, **8** and **10**, while (**C**) view illustrates the 3D superimposition between the original ligand sorafenib (red), **8** (green) and **10** (yellow) within the active site of VEGFR-2 (PDB code: 4ASD), respectively.

Regarding the active site of VEGFR-2 in Figure 7, the coumarin-triazole derivatives **8** and **10** revealed promising energy scores of −9.72 and −10.85 kcal/mol, respectively. The triazole moiety in both **8** and **10** showed arene–cation interaction with Lys868, and the glycoside part displayed H-bond acceptors between the hydroxyl oxygen and the backbone of Cys919 (distance: 2.92, 2.78 Å, respectively). The coumarin core illustrated arene–cation interaction with Leu889 in target **8** and with Cys1045 in analog **10**.

**Figure 8:**
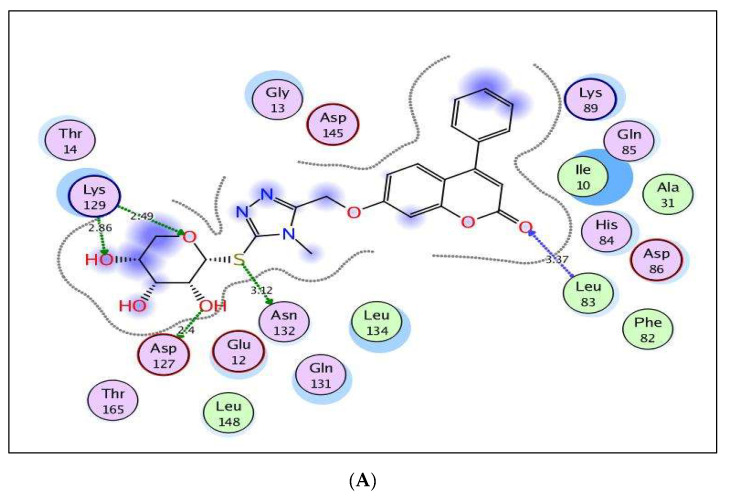

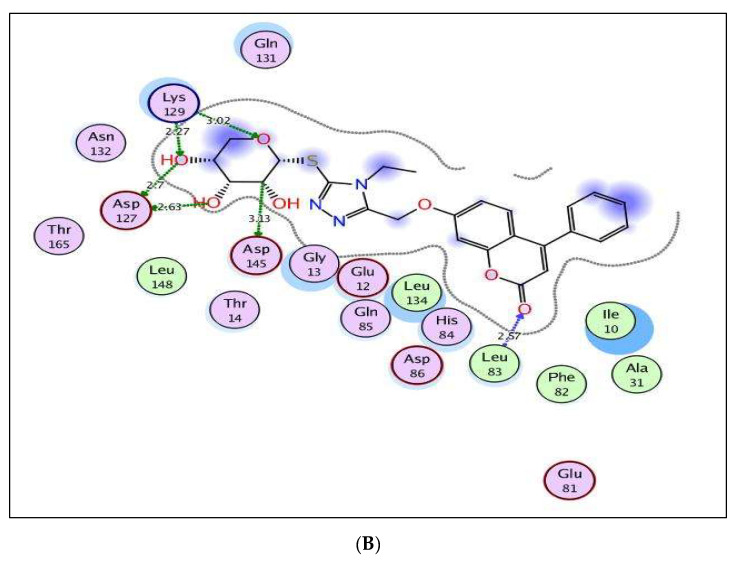

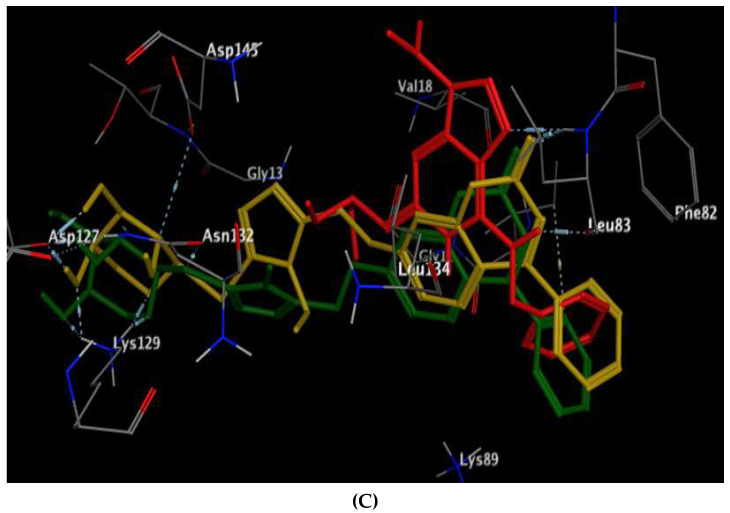
(**A**,**B**) views illustrate the 2D binding features of the coumarin targets, **8** and **10**, while (**C**) view illustrates the 3D superimposition between the original ligand roscovitine (red), **8** (green) and **10** (yellow) within the active site of CDK-2/cyclin A2 (PDB code: 3DDQ), respectively.

The excellent energy scores of coumarin-triazole derivatives **8** and **10** upon docking within the active site of CDK-2/cyclin A2 (−11.28 and −11.70 kcal/mol, respectively), supported the promising inhibitory activity results that was approximately equal or superior to the original ligand, roscovitine. The coumarin nucleus in both targets **8** and **10** was inserted nicely with the binding site through two H-bond acceptors between the carbonyl oxygen at p-2 and the backbone of Leu83 (distance: 3.37, 2.57 Å, respectively) (Figure 8). In addition, the glycoside core shared hydrogen bonding with Asp127 and Lys129 in both analogs **8** and **10**. The fixation of coumarin **8** was synergized through extra H-bond donor between sulfur atom and the sidechain of Asn132 (distance: 3.12 Å). Meanwhile, the existence of ethyl group at p-4 of thioxotriazole moiety instead of methyl in the target **10** gave the chance for H-bond formation between the glycoside part and the sidechain of Asp145 (distance: 3.13 Å).

At the end and depending upon the gained results, it could be concluded that the existence of coumarin-triazole-glycoside hybrid fragments in compounds **8** and **10** gave the chance for more fitting ability within the screened receptors in a similar manner with the original ligands and occupied the same active pockets (superimposition Figure 6C, Figure 7C and Figure 8C). The presence of ethyl group at p-4 of thioxotriazole moiety instead of methyl in the target **10** increases the binding affinity within the active sites of the screened enzymes. In addition, the chain elongation between coumarin and triazole moieties in compound **21** could be the reason for non-fitting in an adequate manner within the three receptors and hence the weak inhibitory activity.

## 3. Experimental

### 3.1. Chemistry

Chemicals were bought from Merck and Sigma-Aldrich which were used without further purification. TLC was performed using aluminum plates pre-coated with silica gel 60 or 60 F254 (Merck) and visualized by iodine or UV light (254 nm). Melting points were determined on a Böetius PHMK (VebAnalytik Dresden) apparatus. The NMR spectra were recorded on a Varian Gemini 300 and Bruker DRX 400 spectrometer at 25 °C, unless otherwise stated. ^1^H- and ^13^C-NMR signals were referenced to TMS and the solvent shift ((CD_3_)_2_SO δH 2.50 and δC 39.5). Coupling constants are given in Hz and without sign. The IR-spectra were recorded by Nexus 670 FT-IR FT-Raman spectrometer using KBr disc. IR, Ms spectra, and the elemental analyses were performed at Micro-Analytical Laboratory, Central Services Laboratory, Faculty of Science, Cairo University, Egypt.

- 4-Phenyl-7-((4-(methyl/ethyl)-5-(1-(*O*-acetyl-*β-D*-glycopyranosyl)thio)-4*H*-1,2,4-triazol-3-yl)methoxy)-2*H*-chromen-2-one (3-6)

*General procedure:* To a solution of triazole-5-thiol derivative 1a,b (10 mmole) in acetone (30 mL) anhydrous potassium carbonate (10 mmole) was added, and the mixture was stirred at room temperature for 1 h, 2,3,4,6-tetra-*O*-acetyl-D-galctopyranosyl bromide or 2,3,4-tri-*O*-acetyl-D-xylopyranosyl (10 m mole) were added and stirred at room temperature until the reaction was complete as assessed by TLC. The solvent was evaporated under reduced pressure at 40 °C, and the residue was washed with distilled water to remove potassium bromide formed. The product was dried and crystallized from ethanol to give the 1,2,4-triazole-thioglycoside products 3–6.

- 4-Phenyl-7-((4-methyl-5-(1-(2,3,4,6-tetra-*O*-acetyl-*β*-D-galactopyranosyl)thio)-4*H*-1,2,4-triazol-3-yl)methoxy)-2*H*-chromen-2-one (3)

Yield: 72%; mp 211–213 °C; IR (KBr) cm^-1^, ν: 2924 (C-H ), 1700–1728 (C=O), 1612 (C=N); ^1^H NMR (DMSO-d_6_) δ/ppm: 1.94, 1.95, 1.96, 1.97 (4s, 12H, 4CH_3_), 3.46 (s, 3H, CH_3_), 3.52 (dd, *J* = 3.2, *J* =10.8 Hz, 1H, H-6′), 3.55 (dd, *J* = 6.8.2, *J* = 10.8 Hz, 1H, H-6′′), 3.64 (m, 1H, H-5′), 4.52 (dd, *J* = 3.8, *J* = 8.8 Hz, 1H, H-4′ ), 4.99 (t, *J* = 8.8 Hz, 1H, H-3′), 5.36 (s, 2H, OCH_2_), 5.42 (dd, *J* = 9.8 Hz, *J* = 8.8 Hz, 1H, H-2′), 5.52 (d, *J* = 9.8 Hz, 1H, H-1′), 6.26 (s, 1H, coumarin-H^3^), 7.02 (s, 1H, Ar-H), 7.25 (d, *J* = 7.8 Hz, 1H, Ar-H), 7.36 (d, *J* = 7.8 Hz, 1H, Ar-H), 7.48 (m, 5H, Ar-H). ^13^C NMR (DMSO-d_6_) δ/ppm: 20.9, 21.0, 21.1 (4CH_3_), 32.9 (CH_3_), 60.7 (CH_2_), 61.7 (C-6′), 64.7 (C-4′), 67.9 (C-2′), 68.8 (C-3′), 70.3 (C-5′), 92.1 (C-1′), 103.0, 112.4, 113.4, 113.5, 128.4, 128.5,129.3, 129.44, 129.4, 135.2, 146.3, 155.7, 160.0, 160.2 (Ar-C, triazole-C^3,5^), 160.8 (coumarin C-2), 170.4, 170.5, 1670.6 (4CO). Anal. calcd. for C_33_H_33_N_3_O_12_S: C, 56.97; H, 4.78; N, 6.04. Found: C, 56.92; H, 4.72; N, 6.09.

- 4-Phenyl-7-((4-methyl-5-(1-(2,3,4-tri-*O*-acetyl-*β*-D-xylopyranosyl)thio)-4*H*-1,2,4-triazol-3-yl)methoxy)-2*H*-chromen-2-one (4)

Yield: 70%; mp 132–133 °C; IR (KBr) cm^−1^, ν: 2920 (C-H ), 1705–1734 (C=O), 1610 (C=N); ^1^H NMR (DMSO-d_6_) δ/ppm: 1.97, 1.98, 2.00 (3s, 9H, 3CH_3_), 3.36 (s, 3H, CH_3_), 3.56 (dd, *J* = 2.8, *J* =10.8 Hz, 1H, H-5′), 3.62 (dd, *J* = 10.8, *J* =7.4 Hz, 1H, H-5′′), 4.92 (dd, *J* = 7.4, *J* = 9.4 Hz, 1H, H-4′), 5.21 (t, *J* = 9.4 Hz, 1H, H-3′), 5.37 (s, 2H, OCH_2_), 5.41 (dd, *J* = 9.8, *J* = 9.2 Hz, 1H, H-2′), 5.62 (d, *J* = 9.8 Hz, 1H, H-1′), 6.27 (s, 1H, coumarin-H^3^), 7.01 (s, 1H, Ar-H), 7.22 (d, *J* = 7.5 Hz, 1H, Ar-H), 7.36 (d, *J* = 7.5 Hz, 1H, Ar-H), 7.34 (m, 5H, Ar-H). ^13^C NMR (DMSO-d6) δ/ppm: 20.8, 21.9, 20.9 (3CH_3_), 33.3 (CH_3_), 57.9 (OCH_2_), 62.1 (C-5′), 67.1 (C-4′), 69.1 (C-2′), 70.9 (C-3′), 96.3 (C-1′), 103.3, 112.3, 112.9, 128.8, 128.9, 129.0, 129.6, 130.2, 130.2, 130.3, 130.3, 141.0, 155.8, 155.9 (Ar-C, triazole-C^3,5^), 160.5 (coumarin C-2), 168.4, 168.5. 168.9 (3CO). Anal. calcd. for C_30_H_29_N_3_O_10_S: C, 57.78; H, 4.69; N, 6.74. Found: C, 57.70; H, 4.63; N, 6.70.

- 4-Phenyl-7-((4-ethyl-5-(1-(2,3,4,6-tetra-*O*-acetyl-*β*-D-galactopyranosyl)thio)-4*H*-1,2,4-triazol-3-yl)methoxy)-2*H*-chromen-2-one (5)

Yield: 72%; mp 151–152 °C; IR (KBr) cm^−1^, ν: 2922 (C-H), 1749 (C=O), 1705 (C=O), 1608 (C=N); ^1^H NMR (DMSO-d_6_) δ/ppm: 1.25 (t, *J* = 6.8 Hz, 3H, CH_3_), 1.96, 1.97, 1.98, 1.99 (4s, 12H, 4CH_3_), 3.90 (m, 2H, H-6′, H-6′′), 4.04 (m, 1H, H-5′), 4.29 (q, *J* = 6.8 Hz, 2H , CH_2_), 5.03 (dd, *J* = 7.2, *J* = 8.2 Hz, 1H, H-4′), 5.29 (t, *J* = 8.2 Hz, 1H,H-3′), 5.38 (s, 2H, OCH_2_), 5.49 (dd, *J* = 8.2, *J* = 9.8 Hz, 1H, H-2′), 5.75 (d, *J* = 9.8 Hz, 1H, H-1′), 6.27 (s, 1H, coumarin-H^3^), 6.98 (s, 1H, Ar-H), 7.27 (d, *J* = 7.5 Hz, 1H, Ar-H), 7.34 (d, *J* = 7.5 Hz, 1H, Ar-H), 7.38 (m, 5H, Ar-H). ^13^C NMR (DMSO-d_6_) δ/ppm: 13.5 (CH_3_), 20.6, 20.7, 20.8, 21.1 (4CH_3_), 33.4, 61.8 (2CH_2_), 63.0 (C-6′), 63.1 (C-4′), 67.4 (C-2′), 69.6 (C-3′), 70.6 (C-5′), 94.3 (C-1′), 102.9, 110.3, 110.4, 128.2, 128.1, 128.3, 129.4, 129.5, 129.6, 132.3, 146.7, 152.5, 152.6, 160.4 (Ar-C, triazole-C^3,5^), 161.5 (coumarin C-2), 168.6, 168.8 169.0, 169.1, 169.4 (5CO). Anal. calcd. for C_34_H_35_N_3_O_12_S: C, 57.54; H, 4.97; N, 5.92. Found: C, 57.61; H, 4.88; N, 5.99.

- 4-Phenyl-7-((4-ethyl-5-(1-(2,3,4-tri-*O*-acetyl-*β*-D-xylopyranosyl)thio)-4*H*-1,2,4-triazol-3-yl)methoxy)-2*H*-chromen-2-one (6)

Yield: 73%; mp 127–128 °C; IR (KBr) cm^−1^, ν: 2923 (C-H), 1735 (C=O), 1719 (C=O); ^1^H NMR (DMSO-d_6_) δ/ppm: 1.25 (t, *J* = 6.8 Hz, 3H, CH_3_), 1.96, 1.98, 1.99 (3s, 9H, 3CH_3_), 3.82 (dd, *J* = 3.8, *J* = 10.8 Hz, 1H, H-5′), 3.99 (dd, *J* = 10.8, *J* = 7.8. Hz, 1H, H-5′′), 4.10 (q, *J* = 6.8 Hz, 2H, CH_2_), 4.81 (dd, *J* = 8.4, *J* = 6.8 Hz, 1H, H-4′), 5.26 (t, *J* = 8.4 Hz, 1H, H-3′), 5.29 (m, 3H, OCH_2_, H-2′), 5.57 (d, *J* = 9.8 Hz, 1H, H-1′), 6.26 (s, 1H, coumarin-H^3^), 6.98 (s, 1H, Ar-H), 7.25 (d, *J* = 7.5 Hz, 1H, Ar-H), 7.36 (d, *J* = 7.5 Hz, 1H, Ar-H), 7.48 (m, 5H, Ar-H). ^13^C NMR (DMSO-d_6_) δ/ppm: 13.8 (CH_3_), 20.8, 20.9, 21.0 (3CH_3_), 31.5, 61.1 (2CH_2_), 68.2 (C-5′), 70.1 (C-4′), 70.2 (C-2′), 71.6(C-3′), 95.1 (C-1′), 102.9, 113.1, 113.4, 128.9, 129.0, 129.4, 129.5, 135.5, 147.8, 155.7, 155.8, 160.3 (Ar-C, triazole-C^3,5^), 160.4 (coumarin C-2), 169.6, 169.6, 170.1 (3CO). Anal. calcd. for C_31_H_31_N_3_O_10_S: C, 58.39; H, 4.90; N, 6.59. Found: C, 58.48; H, 4.99; N, 6.49.

- 4-Phenyl-7-((4-methyl-5-(1-(*β-D*-glycopyranosyl)thio)-4*H*-1,2,4-triazol-3-yl)methoxy)-2*H*-chromen-2-one (7-10)

*General procedure:* The acetylated glycoside derivative (3–6) (0.5 g) was dissolved in a saturated methanolic ammonia (20 mL) with stirring at 0 °C and stirred at the same temperature for 30 minutes and then at room temperature for 6 hours. After completion of the deprotection reaction as indicated by TLC (petroleum ether-hexane, 2:1), the solvent was removed under vacuum at 40 °C and to the residue, diethyl ether (25 mL) was added with vigorous stirring to afford a solid which was filtered, dried and crystallized from cold ethanol affording the glycoside derivatives 7–10, respectively.

- 4-Phenyl-7-((4-methyl-5-(1-(*β*-D-galactopyranosyl)thio)-4*H*-1,2,4-triazol-3-yl)methoxy)-2*H*-chromen-2-one (7)

Yield: 65%; mp 182–183 °C; IR (KBr) cm^−1^, ν: 3414–3390 (OH), 2924 (CH), 1713 (C=O), 1612 (C=N); ^1^H NMR (DMSO-d_6_) δ/ppm: 3.47 (s, 3H, CH_3_), 3.59 (m, 2H, H-6′ H-6′′), 3.85 (m, 1H, H-5′), 4.04 (m, 2H, H-3′, H-4′ ), 4.17 (m, 2H, OH, H-2′), 4.88 (m, 3H, 3OH), 5.37 (s, 2H, OCH_2_), 5.61 (d, *J* = 10.2 Hz, 1H, H-1′), 6.26 (s, 1H, coumarin-H^3^), 7.20 (s, 1H, Ar-H), 7.27 (d, *J* = 7.5 Hz, 1H, Ar-H), 7.36 (d, *J* = 7.5 Hz, 1H, Ar-H), 7.48 (m, 5H, Ar-H). ^13^C NMR (DMSO-d_6_) δ/ppm: 30.7 (CH_3_), 62.3 (OCH_2_), 63.6 (C-6′), 70.5 (C-4′), 70.6 (C-2′), 81.2 C-3′), 86.1 (C-5′), 95.7 (C-1′), 107.9, 108.6, 108,7, 109,6, 109.7, 120.9, 124.2, 129.0, 129.7, 134.3, 146.3, 156.2 (Ar-C, triazole-C^3,5^), 160.0 (coumarin C-2). Anal. Calcd. For C_25_H_25_N_3_O_3_S: C, 56.92; H, 4.78; N, 7.97. Found: C, 56.98; H, 4.71; N, 7.92.

- 4-Phenyl-7-((4-methyl-5-(1-(*β-D*-xylopyranosyl)thio)-4*H*-1,2,4-triazol-3-yl)meth-oxy)-2*H*-chromen-2-one (8)

Yield: 65%; mp 231–232 °C; IR (KBr) cm^−1^, ν: 3424–3395 (OH), 2924 (C-H), 1718 (C=O); ^1^H NMR (DMSO-d_6_) δ/ppm: 3.74 (s, 3H, CH_3_), 3.74 (m, 2H, H-5′, H-5′′), 3.85 (m, 2H, H-3′, H-4′), 4.04 (m, 1H, H-2′), 4.50 (m, 2H, 2OH), 4.88 (m, 1H, OH) 5.37 (s, 2H, OCH_2_), 5.58 (d, *J* = 9.8 Hz, 1H, H-1′), 6.27 (s, 1H, coumarin-H^3^), 7.21 (s, 1H, Ar-H), 7.28 (d, *J* = 7.5 Hz, 1H, Ar-H), 7.35 (d, *J* = 7.5 Hz, 1H, Ar-H), 7.39 (m, 5H, Ar-H). ^13^C NMR (DMSO-d_6_) δ/ppm: 30.7 (CH_3_), 61.8 (OCH_2_), 62.8 (C-5′), 68.4 (C-4′), 73.4 (C-2′), 67.2 C-3′), 98.0 (C-1′), 104.1, 111.8, 112.7, 113.0, 128.4, 128.5, 128.6, 129.0, 129.1, 135.1, 148.1, 155,4, 155,9, 157.3, 160.3 (Ar-C, triazole-C^3,5^), 160.5 (coumarin C-2). Anal. Calcd. For C_24_H_23_N_3_O_7_S: C, 57.94; H, 4.66; N, 8.45; Found: C, 57.90; H, 4.60; N, 8.459.

- 4-Phenyl-7-((4-ethyl-5-(1-(*β-D*-galactopyranosyl)thio)-4*H*-1,2,4-triazol-3-yl)meth-oxy)-2H-chromen-2-one (9)

Yield: 72%; mp 184–185 °C; IR (KBr) cm^−1^, ν: 3419–3405 (OH), 2923 (C-H), 1715 (C=O), 1612 (C=N); ^1^H NMR (DMSO-d_6_) δ/ppm: 1.23 (t, *J* = 6.6 Hz, 3H, CH_3_), 3.43 (m, 2H, H-6′, H-6′′), 3.73 (m, 1H, H-5′), 3.78 (m, 2H, H-3′, H-4′), 3.97 (m, 2H, OH, H-2′), 4.15 (q, *J* = 6.6 Hz, 2H, CH_2_), 4.87 (m, 3H, OH), 5.40 (s, 2H, OCH_2_), 5.81 (d, *J* = 9.8 Hz, 1H, H-1′), 6.13 (s, 1H, coumarin-H^3^), 7.04 (s, 1H, Ar-H), 7.21 (d, *J* = 7.5 Hz, 1H, Ar-H), 7.35 (d, *J* = 7.5 Hz, 1H, Ar-H), 7.49 (m, 5H, Ar-H). ^13^C NMR (DMSO-d_6_) δ/ppm: 13.8 (CH_3_), 31.3 (CH_2_), 60.9 (OCH_2_), 63.64 (C-6′), 69.7 (C-4′), 77.9 (C-2′), 81.9 C-3′), 86.1 (C-5′), 93.9 (C-1′), 103.1, 110.2, 111.7, 127.7, 127.9, 128.2, 128.3, 138.9, 129.4, 135.3, 147.8, 155.8, 159.2, 160.4 (Ar-C, triazole-C^3,5^), 160.5 (C=O). Anal. Calcd. For C_26_H_27_N_3_O_8_S: C, 57.66; H, 5.03; N, 7.76. Found: C, 57.53; H, 5.09; N, 7.82.

- 4-Phenyl-7-((4-ethyl-5-(1-(*β-D*-xylopyranosyl)thio)-4*H*-1,2,4-triazol-3-yl)meth-oxy)-2H-chromen-2-one (10)

Yield: 75%; mp 178–179 °C; IR (KBr) cm^−1^, ν: 3429–3405 (OH), 2920 (C-H), 1716 (C=O); ^1^H NMR (DMSO-d_6_) δ/ppm: 1.23 (t, *J* = 6.8 Hz, 3H, CH_3_), 3.73 (m, 2H, H-5′, H-5′′), 3.75 (m, 1H, H-4′), 3.96 (m, 1H, H-3′), 4.13 (m, 3H, CH_2_, H-2′), 4.97 (m, 3H, 3OH), 5.40 (s, 2H, OCH_2_), 5.59 (d, *J* = 10.2 Hz, 1H, H-1′), 6.13 (s, 1H, coumarin-H^3^), 6.99 (s, 1H, Ar-H), 7.21 (d, *J* = 8.4 Hz, 1H, Ar-H), 7.34 (d, *J* = 8.4 Hz, 1H, Ar-H), 7.49 (m, 5H, Ar-H). Anal. Calcd. For C_25_H_25_N_3_O_7_S: C, 58.70; H, 4.93; N, 8.21. Found: C, 58.79 H, 4.99; N, 8.28.

- Synthesis of disubstituted-1,2,4-triazol compounds 11–14

*General procedure:* To a solution of the triazolyl-coumarin derivative 1a,b (10 mmole) in dry acetonitrile (15 mL), anhydrous potassium carbonate (1.38 g, 10 mmole) was added, and the mixture was stirred at room temperature for 1 hour. 2-(2-chloroethoxy)ethan-1-ol or 3-chloropropane-1,2-diol (10 mmole) was added and stirring was continued for 18 h at 70 °C and then filtered. The solvent was evaporated and the remaining solid material was recrystallized from ethanol to give compounds 11–14, respectively.

- 4-Phenyl-7-((5-((2-(2-hydroxyethoxy)ethyl)thio)-4-methyl-4*H*-1,2,4-triazol-3-yl)methoxy)-2*H*-chromen-2-one (11)

Yield: 65%; mp 155–156 °C; IR (KBr) cm^−1^, ν: 3431 (OH), 2957 (C-H), 1705 (C=O), 1610 (C=N); ^1^H NMR (DMSO-d_6_) δ/ppm: 3.12 (m, 5H, CH_2_, CH_3_), 3.27 (m, 2H, CH_2_), 3.75 (m, 4H, 2CH_2_), 5.42 (s, 2H, OCH_2_), 5.53 (m, 1H, OH), 6.23 (s, 1H, coumarin-H^3^), 7.17 (s, 1H, Ar-H), 7.27 (d, *J* = 8.4 Hz, 1H, Ar-H), 7.33 (d, *J* = 8.4 Hz, 1H, Ar-H), 7.46 (m, 5H, Ar-H). Anal. calcd. for C_23_H_23_N_3_O_7_S: C, 60.91; H, 5.11; N, 9.27. Found: C, 60.99; H, 5.19; N, 9.17.

- 4-Phenyl-7-((4-ethyl-5-((2-(2-hydroxyethoxy)ethyl)thio)-4*H*-1,2,4-triazol-3-yl)methoxy)-2*H*-chromen-2-one (12)

Yield: 65%; mp 152–153 °C; IR (KBr) cm^−1^, ν: 3429 (OH), 2924 (C-H), 1721 (C=O), 1611 (C=N); ^1^H NMR (DMSO-d_6_) δ/ppm: 1.24 (t, *J* = 6.8 Hz, 3H, CH_3_), 3.13 (m, 2H, CH_2_), 3.39 (t, *J* = 7.2 Hz, 2H, CH_2_), 3.49 (t, *J* = 7.4 Hz, 2H, CH_2_), 3.59 (m, 2H, CH_2_), 4.06 (q, *J* = 6.8 Hz, 2H, CH_2_), 5.43 (s, 2H, OCH_2_), 4.97 (m, 1H, OH), 6.23 (s, 1H, coumarin-H^3^), 7.06 (s, 1H, Ar-H), 7.24 (d, *J* = 8.2 Hz, 1H, Ar-H), 7.34 (d, *J* = 8.2 Hz, 1H, Ar-H), 7.43 (m, 5H, Ar-H). ^13^C NMR (DMSO-d_6_) δ/ppm: 15.6 (CH_3_), 32.6, 40.7, 40.8, 60.6, 69.4, 72.7 (6CH_2_), 102.8, 112.1, 113.5, 126.5, 128.3. 128.7, 128.8, 128.9, 129.0, 135.1, 146.7, 151.4, 155.5, 160.4 (Ar-C, triazole-C^3,5^), 161.1 (C=O). Anal. calcd. for C_24_H_25_N_3_O_5_S: C, 61.66; H, 5.39; N, 8.99. Found: C, 61.59; H, 5.33; N, 9.12.

- 4-Phenyl-7-((5-((2,3-dihydroxypropyl)thio)-4-methyl-4*H*-1,2,4-triazol-3-yl)meth-oxy)-2*H*-chromen-2-one (13)

Yield: 65%; mp 170–171 °C; IR (KBr) cm^−1^, ν: 3429 (OH), 2950 (C-H), 1707 (C=O), 1608 (C=N); ^1^H NMR (DMSO-d_6_) δ/ppm: 3.33 (m, 5H, CH_3_, CH_2_), 3.50 (m, 1H, CHOH), 3.65 (m. 2H, CH_2_), 5.01 (m, 2H, 2OH), 5.36 (s, 2H, OCH_2_), 6.21 (s, 1H, coumarin-H^3^), 7.00 (s, 1H, Ar-H), 7.23 (d, *J* = 8.5 Hz, 1H, Ar-H), 7.33 (d, *J* = 8.5 Hz, 1H, Ar-H), 7.48 (m, 5H, Ar-H). Anal. calcd. for C_22_H_21_N_3_O_7_S: C, 60.13; H, 4.82; N, 9.56. Found: C, 60.19; H, 4.80; N, 9.50.

- 4-Phenyl-7-((5-((2,3-dihydroxypropyl)thio)-4-ethyl-*4H*-1,2,4-triazol-3-yl)methoxy)-2*H*-chromen-2-one (14)

Yield: 65%; mp 180–181 °C; IR (KBr) cm^−1^, ν: 3418 (OH), 2923 (C-H), 1716 (C=O), 1605 (C=N); ^1^H NMR (DMSO-d_6_) δ/ppm: 1.29 (t, *J* = 6.8 Hz, 3H, CH_3_), 3.05, 3.18 (dd, 2H, SCH_2_), 3.45 (m, 2H, CH_2_), 3.65 (m, 1H, CHOH), 4.09 (q, *J* = 6.8 Hz, 2H, CH_2_), 5.21 (m, 1H, OH), 5.43 (s, 2H, OCH_2_), 5.56 (m, 1H, OH), 6.23 (s, 1H, coumarin-H^3^), 7.02 (s, 1H, Ar-H), 7.23 (d, *J* = 8.5 Hz, 1H, Ar-H), 7.34 (d, *J* = 8.5 Hz, 1H, Ar-H), 7.48 (m, 5H, Ar-H). ^13^C NMR (DMSO-d_6_) δ/ppm: 15.5 (CH_3_), 37.4, 44.0, 61.7, 68.7 (4CH_2_), 70.6 (*C*H-OH), 105.9, 113.6, 113.7, 128.7, 128.8. 128.9, 129.1, 135.2, 146.8, 151.5, 155.6, 160.5 (Ar-C, triazole-C^3,5^), 161.2 (C=O). Anal. Calcd. for C_23_H_23_N_3_O_5_S: C, 60.91; H, 5.11; N, 9.27. Found: C, 60.99; H, 5.16; N, 9.20.

- 7-(3-Iodopropoxy&4-iodobutoxy )-4-phenyl-2*H*-chromen-2-one (16, 17)

*General procedure:* To a solution of 7-hydroxy-4-phenyl-2*H*-chromen-2-one (15) (2.11 g, 10 mmole) in dry acetonitrile (15 mL) anhydrous potassium carbonate (1.38 g, 10 mmole) was added, and the mixture was stirred at room temperature for 1 h. 1,3-Diiodopropane or 1,4-diiodobutane (30 mmole) were added drop-wise, and stirring was continued for 18 h at 90 °C and then filtered. After filtration, the solvent was evaporated under vacuum, and the remaining residue was recrystallized from ethanol to give compounds 16 or 17, respectively.

- 7-(3-Iodopropoxy)-4-phenyl-2*H*-chromen-2-one (16)

Yield: 76%; mp 130–131 °C; IR (KBr) cm^−1^, ν: 2920 (C-H), 1713 (C=O), 1611 (CN); ^1^H NMR (DMSO-d_6_) δ/ppm: 2.14 (m, 2H, CH_2_), 3.13 (t, *J* = 6.2 Hz, 2H, CH_2_), 4.10 (t, *J* = 6.2 Hz, 2H, CH_2_), 6.28 (s, 1H, coumarin-H^3^), 7.04 (s, 1H, Ar-H), 7.16 (d, *J* = 8.0 Hz, 1H, Ar-H), 7.27 (m, 5H, Ar-H), 7.53 (d, *J* = 8.0 Hz, 1H, Ar-H). ^13^C NMR (DMSO-d_6_) δ/ppm: 4.1, 32.7, 68.6 (3CH_2_), 102.5, 111.6, 111.7, 111.8, 111.9, 1131, 113.2, 1289.3, 129.4, 130.1, 133.4, 155.9, 155.6, 159.0 (Ar-C, triazole-C^3,5^), 160.5 (coumarin C-2). MS m/z: 277.02 406.10 (M^+^, 60.38%). Anal. calcd. for C_18_H_15_IO_3_: C, 53.22; H, 3.72. Found: C, 53.29; H, 3.78.

- 7-(4-Iodobutoxy)-4-phenyl-2*H*-chromen-2-one (17)

Yield: 79%; mp 143–145 °C; IR (KBr) cm^−1^, ν: 2921 (C-H), 1715 (C=O); ^1^H NMR (DMSO-d_6_) δ/ppm: 1.73 (m, 4H, 2CH_2_), 3.17 (t, *J* = 6.8 Hz, 2H, CH_2_), 4.05 (t, *J* = 6.4 Hz, 2H, CH_2_), 6.10 (s, 1H, coumarin-H^3^), 6.93 (s, 1H, Ar-H), 7.24 (m, 5H, Ar-H), 7.41 (d, *J* = 8.8 Hz, 1H, Ar-H), 7.49 (d, *J* = 6.8 Hz, 1H, Ar-H). ^13^C NMR (DMSO-d_6_) δ/ppm: 8.1, 26.0, 28.6, 67.6 (4CH_2_), 104.5, 111.1, 111.2, 126.3, 126,4, 129.3, 133.1, 145.9, 158.2, 158.3, 160.2 (Ar-C, triazole-C^3,5^), 160.3 (C=O). MS m/z: 420.20 (M^+^, 4.71%). Anal. calcd. for C_19_H_17_IO_3_: C, 54.30; H, 4.08; Found: C, 54.16; H, 4.02.

- 7-(3-Azidoalkyloxy)-4-phenyl-2*H*-chromen-2-one (18, 19)

To a solution of 7-(3-iodoalkyloxy)-4-phenyl-2*H*-chromen-2-one (16 or 17) (2.11 g, 10 mmole) in DMF-ethanol-H_2_O mixture (2:6:2, 15 mL) sodium azide (0.78 g, 12 mmole) was added, and the reaction mixture was heated at 60 °C temperature for 4 hours and then filtered. The filtrate was evaporated under reduced pressure and the remaining solid was collected and recrystallized from ethanol to give compounds 18 or 19, respectively.

- 7-(3-Azidopropoxy)-4-phenyl-2*H*-chromen-2-one (18)

Yield: 70%; mp 110–111 °C; IR (KBr) cm^−1^, ν: 2959 (C-H), 1976 (-N_3_), 1705 (C=O), ^1^H NMR (DMSO-d_6_) δ/ppm: 1.40 (t, *J* = 6.6 Hz, 2H, CH_2_), 1.70 (m, 2H, CH_2_), 4.06 (t, *J* = 6.8 Hz, 2H, CH_2_), 6.28 (s, 1H, coumarin-H^3^), 7.04 (s, 1H, Ar-H), 7.16 (d, *J* = 8.0 Hz, 1H, Ar-H), 7.27 (m, 5H, Ar-H), 7.53 (d, *J* = 8.0 Hz, 1H, Ar-H). ^13^C NMR (DMSO-d_6_) δ/ppm: 28.3, 46.8, 67.1 (3CH_2_), 102.1, 110.3, 113.2, 113.4, 127.3, 128.0, 128.3 128.5, 139.0, 145.9, 151.7, 153.3, 160.7 (Ar-C, triazole-C^3,5^), 161.9 (C=O). Anal. calcd. for C_18_H_15_N_3_O_3_: C, 67.28; H, 4.71; N, 13.08. Found: C, 67.12; H, 4.76; N, 12.94.

- 7-(4-Azidobutoxy)-4-phenyl-2*H*-chromen-2-one (19)

Yield: 64%; mp 114–116 °C; IR (KBr) cm^−1^, ν: 2950 (C-H), 1973 (N_3_), 1707 (C=O); ^1^H NMR (DMSO-d_6_) δ/ppm: 1.25 (m, 2H, CH_2_), 1.49 (t, *J* = 6.8 Hz, 2H, CH_2_), 1.75 (m, 2H, CH_2_), 4.06 (t, *J* = 6.4 Hz, 2H, CH_2_), 6.26 (s, 1H, coumarin-H^3^), 7.01 (s, 1H, Ar-H), 7.15 (d, *J* = 8.0 Hz, 1H, Ar-H), 7.37 (m, 5H, Ar-H), 7.53 (d, *J* = 8.0 Hz, 1H, Ar-H). ^13^C NMR (DMSO-d_6_) δ/ppm: 25.1, 25.9, 51.8, 69.6 (4CH_2_), 102.1, 110.3, 113.2, 113.4, 127.3, 128.0, 128.3 128.5, 139.0, 145.9, 151.7, 153.3, 160.7 (Ar-C, triazole-C^3,5^), 161.9 (coumarin C-2). Anal. calcd. for C_19_H_17_N_3_O_3_: C, 68.05; H, 5.11; N,12.53. Found: C, 68.15; H, 5.19; N,12.50

- 4-Phenyl-7-(3-(4-(1-(*O*-acetyl*-β*-D-glycopyranosyl))-1*H*-1,2,3-triazol-1-yl)propoxy & butoxy)-2*H*-chromen-2-one (21-23)

To a solution of the azide derivative 18 or 19 (2.0 mmol) in butanol, the terminal acetylenic pyranosyl sugar; namely, 2,3,4,6-tetra-*O*-acetyl-D-galctopyranosyl or 2,3,4-tri-*O*-acetyl-D-xylopyranosyl azide (2 mmol) was added. Sodium ascorbate (0.4 mmol, 0.08 g) and few drops of diisopropylethylamine (DIPEA) followed by CuSO_4_.5H_2_O (0.4 mmol, 0.11 g) were then added separately. The mixture was stirred in the dark at room temperature overnight [(TLC judged; pet. ether: ethyl acetate (4:1)]. Ethyl acetate was then added, and the organic layer was then speared after shaking the mixture twice for five minutes_._ The organic layers were then collected and then dried (Na_2_SO_4_) and evaporated. Further purification by column chromatography [hexane: ethyl acetate (5:1) as eluent] gave the title products.

- 4-Phenyl-7-(3-(4-(1-(2,3,4,6-tetra-*O*-acetyl-*β*-D-galactopyranosyl))-1*H*-1,2,3-triazol-1-yl)propoxy)-2*H*-chromen-2-one (21)

Yield: 77%; mp 114–115 °C; IR (KBr) cm^−1^, ν: 2927 (C-H), 1710 (C=O), 1752 (C=O); ^1^H NMR (DMSO-d_6_) δ/ppm: 1.93, 194, 1.95. 1.97 (4s, 12H, 4CH_3_), 1.99 (m, 2H, CH_2_), 3.34 (t, *J* = 6.8 Hz, 2H, CH_2_), 3.51 (t, *J* = 6.8 Hz, 2H, CH_2_), 3.97 (dd, 1H, *J* = 2.8, *J* = 10.8Hz, H-6′), 4.05 (dd, 1H, *J* = 10.8, *J* = 7.8 Hz, H-6′′), 4.33 (m, 2H, H-5′, H-4′), 4.77 (m, 2H, CH_2_), 5.12 (dd, *J* = 8.8, *J* = 10.2 Hz, 1H, H-2′), 5.49 (t, J = 8.8Hz, 1H, H-3′), 6.03 (d, *J* = 10.2 Hz, 1H, H-1′), 6.13 (s, 1H, coumarin-H^3^), 6.97 (m, 2H, Ar-H), 7.15 (m, 5H, Ar-H ), 7.51 (s, 1H, traizole), 7.59 (d, *J* = 8.2 Hz, 1H, Ar-H). Anal. calcd. for C_35_H_37_N_3_O_13_: C, 59.40; H, 5.27; N, 5.94. Found: C, 59.23; H, 5.33; N, 6.02.

- 4-Phenyl-7-(3-(4-(1-(2,3,4–tria-*O*-acetyl-*β*-D-xylopyranosyl))-1*H*-1,2,3-triazol-1-yl)propoxy)-2*H*-chromen-2-one (22)

Yield: 76%; mp 127–128 °C; IR (KBr) cm^−1^, ν: 2924 (C-H), 1710 (C=O), 1752 (C=O), 1610 (C=N); ^1^H NMR (DMSO-d_6_) δ/ppm: 1.93, 1.96, 1.98 (3s, 9H, 3CH_3_), 2.02 (m, 2H, CH_2_), 3.51 (t, *J* = 6.6 Hz, 2H, CH_2_), 3.54 (t, *J* = 6.6 Hz, 2H, CH_2_), 4.05 (dd, 1H, *J* = 3.4, *J* = 10.6 Hz, H-5′), 4.20 (dd, 1H, *J* = 10.4.4, *J* = 7.8 Hz, H-5′′), 4.42 (dd, 1H, *J* = 7.8, *J* = 8.4 Hz, H-4′), 5.70 (m, 3H, CH_2_, H-2′), 5.14 (dd, *J* = 7.8, *J* = 8.8 Hz, 1H, H-3′), 6.00 (d, *J* = 3.4, *J* = 10.8 Hz, 1H, H-1′), 6.16 (s, 1H, coumarin-H^3^), 6.70 (m, 2H, Ar-H), 7.05 (m, 2H, Ar-H), 7.24 (m, 2H, Ar-H), 7.49 (s, 1H, traizole), 7.59 (m, 2H, Ar-H). ^13^C NMR (DMSO-d_6_) δ/ppm: 20.8, 20.9, 21.0 (3CH_3_), 32.3, 35.1, 56.3 (3CH_2_), 62.0 (C-5′), 69.0 (CH_2_), 69.1 (C-4′), 71.6 (C-2′), 71.7 (C-3′), 98.9 (C-1′), 102.1, 103.2, 110.7, 110.8, 113.1, 128.8, 128.7,128.8, 129.0, 129.2, 129.3, 129.4, 130.2, 155.94, 155.9, 160.4 (Ar-C, traizol-C^4,5^), 160.5 (coumarin-C^2^), 170.0, 170.1 (3C=O). Anal. calcd. for C_32_H_33_N_3_O_11_: C, 60.47; H, 5.23; N, 6.61. Found: C, 60.29; H, 5.18; N, 6.87.

- 4-Phenyl-7-(3-(4-(1-(2,3,4,6-tetra-*O*-acetyl-*β*-D-galactopyranosyl))-1*H*-1,2,3-triazol-1-yl)butoxy)-2*H*-chromen-2-one (23)

Yield: 77%; mp 118–119 °C; IR (KBr) cm^−1^, ν: 2924 (C-H), 1710 (C=O), 1756 (C=O), 1612 (C=N); ^1^H NMR (DMSO-d_6_) δ/ppm: 1.10 (m, 2H, CH_2_), 1.67 (m, 2H, CH_2_), 1.90, 1.93, 1.95, 1.97 (4s 12H, 4CH_3_), 2.04 (m, 2H, CH_2_), 3.99 (dd, *J* = 2.8, *J* = 10.6 Hz, 1H, H-6′), 4.01 (t, *J* = 6.8 Hz, 2H, CH_2_), 4.06 (dd, 1H, *J* = 10.6, *J* = 7.6 Hz, H-6′′), 4.27 (dd, *J* = 7.6, *J* = 8.0 Hz, 1H, H-5′) 4.35 (dd, *J* = 8.0, *J* = 7.8 Hz, 1H, H-4′), 4.74 (m, 2H, CH_2_), 4.84 (dd, *J* = 8.2, *J* = 10.2 Hz, 1H, H-2′), 5.16 (t, *J* = 8.2 Hz, 1H, H-3′), 6.12 (d, *J* = 10.2 Hz, 1H, H-1′), 6.21 (s, 1H, coumarin-H^3^), 6.97 (m, 2H, Ar-H), 7.16 (m, 5H, Ar-H), 7.51 (s, 1H, traizole), 7.61 (d, *J* = 8.4 Hz, 1H, Ar-H). ). ^13^C NMR (DMSO-d_6_) δ/ppm: 30.7, 20.8, 20.9, 21.0 (3CH_3_), 25.5, 26.4, 56.4, 62.1 (4CH_2_), 62.2 (C-6′), 67.9 (CH_2_), 68.0 (C-4′), 68.1 (C-3′), 71.3 (C-2′), 72.5 (C-5′), 97.4 (C-1′), 102.1, 111.7, 113.1, 113.3, 127.8, 128.0, 128.3, 128.8, 128.9, 135.5, 155.7, 156.4, 160.5 (Ar-C, triazol-C^3^,C^5^), 161.3 (coumarin-C^2^), 170.5, 179.6, 170.7 (4CO). Anal. calcd. for C_36_H_39_N_3_O_13_: C, 59.91; H, 5.45; N, 5.82. Found: C, 60.07; H, 5.37; N, 5.73.

- 4-Phenyl-7-(3-(4-(1-(*-β*-D-glycopyranosyl))-1*H*-1,2,3-triazol-1-yl)alkyloxy)-2*H*-chromen-2-one (24-26)

The *O*-acetylated-1,2,3-triazolyl-glycopyranosyl compounds (21–23) (5 mmol) were added portion-wise while stirring in dry methanol saturated with gaseous ammonia (20 mL) at 0 °C. The reaction mixture was stirred for 30 minutes at 0 °C, then for further 7 hours at room temperature. After completion of the deacetylation process (TLC: petroleum ether-hexane, 2:1), the solvent was evaporated under reduced pressure at 40 °C and to the residue, cold diethyl ether (25 mL) was added with vigorous shaking until it affords a solid product, which was filtered and dried and recrystallized from ethanol.

- 4-Phenyl-7-(3-(4-(1-(*β*-D-galactopyranosyl))-1*H*-1,2,3-triazol-1-yl)propoxy)-2*H*-chromen-2-one (24).

Yield: 80%; mp 190–191 °C; IR (KBr) cm^−1^, ν: 3418-3402 (OH), 2923 (C-H), 1716 (C=O); ^1^H NMR (DMSO-d_6_) δ/ppm: 1.82 (m, 2H, CH_2_), 2..45 (t, *J* = 6.4 Hz, 2H, CH_2_), 3.34 (m, 2H, H-6′ H-6′′), 3.51 (m, 1H, H-5′), 4.10 (m, 1H, H-H-4′), 4.21 (t, *J* = 6.6 Hz, 2H ,CH_2_), 4.35 (m, 2H, H-2′, OH), 4.65 (m, 3H, CH_2_, OH), 4.96 (m, 1H, OH), 5.13 (m, 1H, OH), 5.49 (t, *J* = 8.8 Hz, 1H, H-3′), 6.05 (d, *J* = 9.8 Hz, 1H, H-1′), 6.19 (s, 1H, coumarin-H^3^), 6.89 (m, 2H, Ar-H), 7.22 (m, 5H, Ar-H), 7.51 (s, 1H, traizole), 7.61 (d, *J* = 8.4 Hz, 1H, Ar-H). ^13^C NMR (DMSO-d_6_) δ/ppm: 29.0, 32.4 (2CH_2_), 61.6 (C-6′), 66.1, 66.2 (2CH_2_), 70.5 (C-4′), 73.7 (C-3′), 77.1 (C-2′), 77.7 (C-5′), 97.8 (C-1′), 110.8, 110.8, 113.7, 113.7, 126.3, 128.8, 128.9, 129.2, 129.3, 129.5, 130.0, 156.2,160.9 (Ar-C, triazol-C^3,5^) 161.8 (coumarin-C^2^); Anal. calcd. for C_27_H_29_N_3_O_9_: C, 60.11; H, 5.42; N, 7.79. Found: C, 60. 23; H, 5.36; N, 7.88

- 4-Phenyl-7-(3-(4-(1-(*β*-D-xylopyranosyl))-1*H*-1,2,3-triazol-1-yl)propoxy)-2*H*-chromen-2-one (25)

Yield: 79%; mp 188–189 °C; IR (KBr) cm^−1^, ν: 3431-3420 (OH) 2936 (C-H), 1717 (C=O); ^1^H NMR (DMSO-d_6_) δ/ppm: 1.79 (m, 2H, CH_2_), 2.22 (t, *J* = 6.4 Hz, 2H, CH_2_), 3.59 (m, 2H, H-5′, H-5′′), 4.16 (m, 1H, H-4′), 4.25 (t, *J* = 6.6 Hz, 2H, CH_2_), 4.34 (m, 1H, OH), 4.55 (m, 1H, H-2′), 4.70 (m, 3H, CH_2_, OH), 5.01 (m, 1H, OH), 5.48 (t, *J* = 8.6 Hz, 1H, H-3′), 5.99 (d, *J* = 9.8 Hz, 1H, H-1′), 6.19 (s, 1H, coumarin-H_3_), 7.12 (m, 2H, Ar-H), 7.20 (m, 5H, Ar-H ), 7.46 (s, 1H, traizole), 7.88 (d, *J* = 8.4 Hz, 1H, Ar-H). ^13^C NMR (DMSO-d_6_) δ/ppm: 31.3, 36.4, 55.6 (3CH_2_), 66.1 (C-5′), 69.8 (CH_2_), 69.9 (C-4′), 73.4 (C-3′),76.9 (C-2′), 102.1 (C-1′), 102.9, 110.7,110.8, 113.1, 122.0, 128.7, 128.8, 128.9, 129.3, 129.4, 135.4, 130.2, 156.0, 160.6 (Ar-C, triazol-C^3,5^), 161.7 (coumarin-C^2^). Anal. calcd. for C_26_H_27_N_3_O_8_: C, 61.29; H, 5.34; N, 8.25. Found: C, 61.52; H, 5.27; N, 8.16

- 4-Phenyl-7-(3-(4-(1-(*β-D-*galactopyranosyl))-1*H*-1,2,3-triazol-1-yl)butoxy)-2*H*-chromen-2-one (26)

Yield: 78%; mp 195–196 °C; IR (KBr) cm^−1^, ν: 3433-3418 (OH), 2932 (C-H), 1715 (C=O), 1607 (C=N); ^1^H NMR (DMSO-d_6_) δ/ppm: 1.60 (m, 4H, 2CH_2_), 2.45 (t, *J* = 6.8 Hz, 2H, CH_2_), 3.38 (m, 2H, H-6′, H-6′′), 3.61 (m, 1H, H-5′), 3.98 (m, 1H, H-4′), 4.22 (t, *J* = 6.2 Hz, 2H, CH_2_), 4.34 (m, 1H, OH), 4.57 (m, 1H, H-2′), 4.72 (m, 3H, CH_2_, OH), 5.01 (m, 2H, 2OH), 5.30 (t, *J* = 8.8 Hz, 1H, H-3′), 6.03 (d, *J* = 10.2 Hz, 1H, H-1′), 6.17 (s, 1H, coumarin-H^3^), 7.03 (s, 1H, Ar-H), 7.13 (d, *J* = 8.4 Hz, 1H, Ar-H), 7.38 (m, 5H, Ar-H), 7.52 (s, 1H, traizole), 7.62 (d, *J* = 8.4 Hz, 1H, Ar-H). Anal. calcd. for C_28_H_31_N_3_O_9_: C, 60.75; H, 5.64; N, 7.59. Found: C, 60.57; H, 5.57; N, 7.68.

### 3.2. Biological Evaluation

#### 3.2.1. Cytotoxicity Assay

##### Cell Lines

Human osteosarcoma (HOS cell line), human breast adenocarcinoma (MDA-MB-231cell lines), human breast cancer (MCF-7 cell lines), human colorectal adenocarcinoma (Caco2 cell lines), human colon cell line (HCT-116 cell lines), normal human cell lines (BJ-1), and “a telomerase immortalized normal foreskin fibroblast cell line” were obtained from Karolinska Center, Department of Oncology and Pathology, Karolinska Institute and Hospital, Stockholm, Sweden. 

Following culturing for 10 days, the cells were seeded at concentration of 10 × 10^3^ cells per well in case of MCF-7 & MDA, 20 × 10^3^ cells/well in a fresh complete growth medium in case of HCT-116, Caco2, & HOS and 40 × 10^3^ cells/well in case of BJ-1 at 37 °C for 24 h in a water jacketed carbon dioxide incubator. After 48 h’ incubation, the medium was aspirated, and then 40 μl MTT salt (2.5 mg/mL) were added for a further 4 h; 200 μl 10% sodium dodecyl sulphate (SDS) in deionized water were added to each well and incubated overnight at 37 °C. The absorbance was measured at 595 nm with reference 690 nm. Doxorubicin was used as positive control, and 0.5% DMSO was used as negative control [52,53]. IC_50_ values were calculated, using a probit analysis, and by utilizing the SPSS computer program (SPSS for Windows, statistical analysis software package/version 9/1989 SPSS Inc., Chicago, IL, USA).

#### 3.2.2. Cell Cycle Analysis and Apoptosis Detection

Cell cycle analysis and apoptosis detection were carried out by flow cytometry. MCF-7 cells were seeded at 1–5 × 10^4^ and incubated at 37 °C, 5% CO_2_ overnight. After treatment with the tested compound **10**, for 24 h, cell pellets were collected and centrifuged (300× g, 5 min). For cell cycle analysis, cell pellets were fixed with 70% ethanol on ice for 15 min and collected again [54]. The collected pellets were incubated with propidium iodide (PI) staining solution at room temperature for 1 h. Apoptosis detection was performed by Annexin V-FITC apoptosis detection kit (BioVision, Inc, Milpitas, CA, USA) following the manufacturer’s protocol. The samples were analyzed using FACS Calibur flow cytometer (BD Biosciences, San Jose, CA).

#### 3.2.3. Human CASP-7 (Caspase-7) Estimation

The micro-ELISA plate was provided in this kit pre-coated with a CASP-7-specific antibody. A biotinylated CASP-7 antibody and Avidin–horseradish peroxidase (HRP) conjugate were added. Get rid of the excess components. The substrate solution was added. Wells that contain CASP-7, biotinylated detection antibody, and Avidin-HRP conjugate will appear blue in color. The color turns yellow following the addition of sulfuric acid solution. The optical density (OD) was measured at a wavelength of 450 nm ±2 nm [55].

#### 3.2.4. Human Cytochrome c Estimation

Cytochrome c specific antibodies have been precoated onto 96-well plates. Cytochrome c were analyzed by enzyme-linked immunosorbent assays (ELISAs) using commercial kits (ab119521; Abcam, Cambridge, MA, USA). The optimal dilutions were determined, and ELISAs were performed according to the manufacturer’s instructions [56].

#### 3.2.5. Measurement of Bcl-2 Levels

Bcl-2 (ng/mL) in the samples and standards were estimated as reported [57]. A biotin-conjugated antibody was added followed by streptavidin-HRP. The reaction is then terminated by adding acid and absorbance was measured at 450 nm.

#### 3.2.6. Measurement of Bax Levels

Bax protein levels picogram per ml (Pg/mL) were evaluated according to previously reported procedure [58]. Monoclonal antibody, specific to Bax captured on the plate, was added. After incubation, Streptavidin conjugated to Horseradish peroxidase was added. The reaction was then terminated by adding acid and optical density of the color produced measured at 450 nm.

#### 3.2.7. In Vitro Kinase Inhibitory Assessment against EGFR, VEGFR-2 and CDK-2

The promising compounds **8**, **10** and **21** were further examined for their inhibitory activities against EGFR, VEGFR-2 and CDK-2/cyclin A2 [43,44,45]. 

EGFR assay: The master mixture (6 μL 5X Kinase Buffer + 1 μL ATP (500 μM) + 1 μL 50 X PTK substrate + 17 μL water) was prepared, then, 25 μL was added to every well. In addition, 5 μL of Inhibitor solution of each well labeled as “Test Inhibitor” were added. However, for the “Positive Control" and “Blank”, 5 μL of the same solution without inhibitor (Inhibitor buffer) were added. Furthermore, 3 mL of 1X Kinase Buffer by mixing 600 μL of 5X Kinase Buffer with 2400 μL water were prepared. Thus, 3 mL of 1X Kinase Buffer became sufficient for 100 reactions. To the wells designated as “Blank”, 20 μL of 1X Kinase Buffer were added. EGFR enzyme on ice was thawed. Upon first thaw, briefly the tube containing enzyme was spun to recover full content of the tube. The amount of EGFR required for the assay and dilute enzyme to 1 ng/μL with 1X Kinase Buffer was calculated. Moreover, the remaining undiluted enzyme in aliquots was stored at −80 °C. The reaction was initiated by adding 20 μL of diluted EGFR enzyme to the wells designated “Positive Control” and "Test Inhibitor Control"; after that, it was incubated at 30 °C for 40 min. After the 40 minutes’ reaction, 50 μL of Kinase-Glo Max reagent were added to each well, and the plate was covered with aluminum foil and incubated at room temperature for 15 min. Luminescence was measured using the microplate reader.

VEGFR-2 assay: In addition, the effect of the most promising cytotoxic compounds **8**, **10** and **21** on the level of VEGFR-2 in human breast cancer cell line MCF-7 was determined. The cells in culture medium were treated with 20 μL of IC_50_ values of the compounds dissolved in DMSO, then incubated for 24 h at 37 °C, in a humidified 5% CO_2_ atmosphere. The cells were harvested, and the homogenates were prepared in saline using a tight pestle homogenizer until complete cell disruption. The kit uses a double-antibody sandwich enzyme-linked immunosorbent assay (ELISA) to determine the level of human VEGFR-2 in samples. A monoclonal antibody for VEGFR-2 was pre-coated onto 96-well plates. The test samples are added to the wells, and a biotinylated detection polyclonal antibody from goat specific for VEGFR-2 was added subsequently followed by washing with PBS buffer. Avidin-Biotin-Peroxidase complex was added, and the unbound conjugates were washed away with PBS buffer. HRP substrate TMB was used to visualize HRP enzymatic reaction. TMB was catalyzed by HRP to produce a blue color product that changed into yellow after adding acidic stop solution. The density of yellow color is proportional to the human VEGFR-2 amount of the sample captured in the plate. The chroma of color and the concentration of the human VEGFR-2 of the samples were positively correlated, and the optical density was determined at 450 nm. The level of human VEGFR-2 in samples was calculated (pg/ml) as duplicate determinations from the standard curve. Percent inhibition was calculated in comparison to control untreated cells.

Assay of CDK-2/cyclin A2 was achieved through ELISA using an affinity tag labeled capture antibody. Adding the samples and the standard to the wells has been done after that addition of the antibody mix. After the incubation period, the unrestrained substance has been discarded, and the wells have been washed. The added TMB (3,3′,5,5′-tetramethylbenzidine) substrate was prompted by Horseradish peroxidase (HRP), and blue coloration appeared. This reaction was stopped through the addition of stop solution to complete changing in colour from blue to yellow. The created signals were equivalent to the quantity of bound analyte, and the Robonik P2000 ELISA reader was used to record the intensity at a certain wavelength (450 nm). The concentrations of the screened compounds have been calculated through the plotted curve.

### 3.3. Molecular M7odelling Study upon EGFR, VEGFR-2 and CDK-2

The 2D structure of the newly synthesized derivatives **8** and **10** was drawn through ChemDraw. The protonated 3D was employed using standard bond lengths and angles, using Molecular Operating Environment (MOE-Dock) software version 2014.0901 [49,50,51]. Then, the geometry optimization and energy minimization were applied to get the Conf Search module in MOE, followed by saving of the moe file for the upcoming docking process. The co-crystallized structures of EGFR, VEGFR-2 and CDK-2/cyclin A2 kinases with their ligands erlotinib, sorafenib and roscovitine were downloaded (PDB codes: 1M17, 4ASD and 3DDQ, respectively) [45,46,47,48] from the protein data bank. All minimizations were performed using MOE until an RMSD gradient of 0.05 kcal∙mol^−1^Å^−1^ with the MMFF94x force field and the partial charges were automatically calculated. Preparation of the enzyme structures was done for molecular docking using Protonate 3D protocol with the default options in MOE. The London dG scoring function and Triangle Matcher placement method were used in the docking protocol. At first, validation of the docking processes was established by docking of the native ligands, followed by docking of the derivatives **8** and **10** within the ATP-binding sites after elimination of the co-crystallized ligands.

## 4. Conclusions

New derivatives of 1,2,3-triazole-coumarin-glycosyl hybrids and their 1,2,4-triazole-thioglycosidic analogues were designed and efficiently synthesized. The anticancer activity results against the human HOS, MDA-MB-231, MCF-7, Caco2 and HCT-116 cancer cell lines revealed the high potency of the coumarin-triazole-glycoside hybrids **8**, **10** and **21** against MCF-7 and the derivative 16 upon HOS and MDA human cancer cells. For detection of the possible mechanism of action, the inhibitory activity of the promising coumarin derivatives **8**, **10** and **21** was studied against EGFR, VEGFR-2 and CDK-2/cyclin A2 kinases. The coumarin-triazoles **8** and **10** illustrated excellent broad inhibitory activity (IC_50_ of compound **8** = 0.22, 0.93 and 0.24 μM, respectively), (IC_50_ of compound **10** = 0.12, 0.79 and 0.15 μM, respectively), in comparison with the reference drugs, erlotinib, sorafenib and roscovitine, (IC_50_ = 0.18, 1.58 and 0.46 μM, respectively). The effect of the coumarin-1,2,4-triazole-thioglycoside hybrid **10** upon levels of cytochrome c, Bcl-2, Bax, and caspase-7 along with cell cycle analysis revealed upregulation of pro-apoptotic Bax protein, Bax/Bcl-2 ratio, cytochrome c and caspase-7, and downregulation of the anti-apoptotic Bcl-2 protein with cell cycle arrest at S phase. The molecular docking simulation confirmed the promising enzymatic results and outlined the good binding patterns and the well-fitting behaviour of compounds **8** and **10** in the active sites of EGFR, VEGFR-2 and CDK-2/cyclin A2 kinases. The study may efficiently promote future research achievements in the field of drug candidate discovery for cancer treatment.

## Figures and Tables

**Table 1:** In vitro screening of the antiproliferative activities of the compounds against HOS, MDA-MB-231, MCF-7, Caco-2, and HCT-116 cancer cell lines. Preliminary concentration for screening was 100 µM for 48 h; each result is a mean of three replicate samples and values are represented as % inhibition, ± represents standard deviation.

Cytotoxicity % at 100 µM					
Compound	HOS	MDA	MCF-7	Caco-2	HCT-116
**1a**	42.5 ± 1.2	24.1 ± 1.4	15.6 ± 0.2	34.8 ± 1.3	16.8 ± 0.1
**1b**	92 ± 2.1	69.7 ± 2.4	88 ± 1.4	77.7 ± 0.8	81 ± 1.2
**3**	18.4 ± 0.7	15.4 ± 0.5	9.8 ± 0.6	23.2 ± 1.1	11.1 ± 0.4
**4**	45.5 ± 0.6	36.9 ± 1.2	17.1 ± 0.3	24.9 ± 0.6	15 ± 0.6
**5**	24.9 ± 0.6	18.5 ± 0.6	2.8 ± 0.2	20.6 ± 0.3	7.6 ± 0.2
**6**	33.1 ± 0.2	7.4 ± 0.3	14.3 ± 0.1	30.1 ± 0.1	18.9 ± 0.6
**7**	7.3 ± 0.3	15.5 ± 1.1	14.3 ± 0.2	35.7 ± 0.3	16.6 ± 0.8
**8**	41.9 ± 0.7	37.1 ± 2.3	60.1 ± 0.7	28.4 ± 0.6	23 ± 0.6
**9**	20.4 ± 0.4	27.7 ± 0.4	37.8 ± 0.3	1.7 ± 0.1	22.7 ± 0.9
**10**	5.3 ± 0.2	20.6 ± 0.5	66.3 ± 1	0.7 ± 0.003	27.2 ± 0.7
**11**	39.7 ± 0.8	62.4 ± 0.7	86.7 ± 0.7	19.1 ± 0.4	91.7 ± 1.1
**12**	97.2 ± 1.3	91.2 ± 0.8	97.6 ± 0.9	92 ± 1.3	84.4 ± 0.8
**13**	2.8 ± 0.2	0 ± 0.01	51.9 ± 0.2	16.6 ± 0.7	26.1 ± 0.3
**14**	90.5 ± 1.1	97.8 ± 0.7	98.1 ± 0.3	91.2 ± 2.1	95.2 ± 0.9
**15**	62.7 ± 1.5	34.5 ± 0.4	36.8 ± 1.1	46.1 ± 0.6	89.3 ± 0.7
**16**	91.2 ± 2.2	88.3 ± 1.4	35.6 ± 0.8	54.3 ± 0.4	12.8 ± 1.1
**17**	50.8 ± 0.7	7.7 ± 0.4	38.5 ± 1.2	34.3 ± 0.3	38.2 ± 0.7
**21**	58.3 ± 1.7	26.4 ± 0.3	85.4 ± 1.8	59.2 ± 1.1	64.9 ± 0.5
**22**	90.6 ± 1.5	97.1 ± 1.1	91.9 ± 0.8	57.5 ± 0.5	97.3 ± 0.8
**23**	0 ± 0.001	28.6 ± 0.2	35.2 ± 0.3	13.6 ± 0.4	30.4 ± 0.7
**24**	97.6 ± 1.3	97.7 ± 0.8	88.2 ± 1.1	86.5 ± 0.9	99.1 ± 0.8
**25**	90.4 ± 0.8	94.2 ± 2.2	81.9 ± 0.9	82.7 ± 1.1	97.1 ± 1.1
**26**	66.9 ± 2.2	31.4 ± 0.5	91.8 ± 1.2	2.6 ± 0.2	83.9 ± 0.9

**Table 2:** IC_50_ values (µM) of active coumarin derivatives **8**, **10**, **15**, **16** and **21** having ≥60% cytotoxicity over their corresponding cancer cell lines.

IC_50_ (µM)				
Compound	HOS	MDA	MCF-7	HCT-116
**8**	---	---	52.2 ± 1.3	---
**10**	---	---	19.6 ± 0.7	---
**15**	88.3 ± 2.2	---	---	46 ± 2.2
**16**	26.2 ± 1.4	39.5 ± 0.9	---	---
**21**	---	---	38.9 ± 0.8	82.8 ± 2.3

Where ---: not detected, ±: represents standard deviation.

**Table 3:** Kinase inhibitory assessment of the promising derivatives **8**, **10** and **21** against EGFR, VEGFR-2 and CDK-2/cyclinA2 comparing with erlotinib, sorafenib and roscovitine, respectively.

Compound No.	IC_50_ (Mean ± SEM) (µM)		
	EGFR	VEGFR-2	CDK-2/Cyclin A2
**Erlotinib**	0.18 ± 0.05	-	-
**Sorafenib**	-	1.58 ± 0.11	-
**Roscovitine**	-	-	0.46 ± 0.30
**8**	0.22 ± 0.01	0.93 ± 0.42	0.24 ± 0.20
**10**	0.12 ± 0.50	0.79 ± 0.14	0.15± 0. 60
**21**	123 ± 0.10	172 ± 0.02	211± 0.05

IC_50_: Compound concentration necessary to inhibit the enzyme activity by 50%, SEM: Standard error mean; each value is the mean of three values, (-) not detected.

## Data Availability

Not applicable.
